# Executive functioning as a moderator of flossing behaviour among young adults: a temporal self-regulation theory perspective

**DOI:** 10.1080/21642850.2023.2249972

**Published:** 2023-08-27

**Authors:** Adam A. Rogers, Anne E. M. Halvari, Jan-Are K. Johnsen, Anne-Kristin Solbakk

**Affiliations:** a Institute of Clinical Dentistry, University of Oslo, Oslo, Norway; b Institute of Clinical Dentistry, UiT The Arctic University of Norway, Tromso, Norway; c RITMO Centre for Interdisciplinary Studies in Rhythm, Time, and Motion, Department of Psychology, University of Oslo, Oslo, Norway

**Keywords:** Oral hygiene behaviour, executive functioning, temporal self-regulation theory, self-regulation, BRIEF-A

## Abstract

**Background:**

Flossing among young adults is often infrequent and barriers not completely understood. One explanation concerns the capacity for executive functioning (EF) during the self-regulation of behaviour.

**Methods:**

Using Temporal Self-Regulation Theory (TST) as a framework to explore EF, young adults from Norwegian universities completed a survey that measured monthly flossing frequency, flossing-related intentions and behavioural prepotency (BP), and EF using the Behaviour Rating Inventory of Executive Function – Adult Version (BRIEF-A).

**Results:**

Data from 362 participants were analysed. The TST-model explained a substantial proportion of variance in monthly flossing (*R*^2^ = 0*.*74), and flossing was associated directly with intention and BP, and interactions between intention and both BP and global-EF. Sub-domains of EF were explored using the same model, revealing that behavioural regulation processes, specifically those related to emotional control and shifting between tasks, offered better fit. Simple slopes revealed that moderation effects were only present at lower levels of BP.

**Conclusion:**

EF plays a role in moderating the translation of intentions into flossing behaviour. Specifically, emotional control and task-shifting appear to be influential, and this influence increases when habitual and environmental support (i.e. BP) is reduced. Overcoming EF-barriers may represent a key step in establishing flossing behaviours.

## Introduction

### Background

The accumulation of intra-oral plaque around the teeth and gums is a primary risk factor for dental disease. While tooth-brushing represents an effective method of plaque removal (Kumar et al., 2016), it is also recommended to remove plaque from between the teeth; areas where toothbrush-access is limited. Among individuals with healthy gums and small interdental spaces, the ideal method of removing interdental plaque is by using dental floss (Chapple et al., 2015). Flossing reduces the accumulation of plaque beyond the effect provided by tooth-brushing alone (Worthington et al., 2019) and is recognised as an important part of daily oral hygiene (Vernon et al., 2017).

One demographic cohort that may benefit from improved flossing behaviour is young adults. Young adulthood is identified as a key risk-period for dental disease (Kassebaum et al., 2015), and a critical time for establishing interdental cleaning behaviours that prevent gum disease in later life (Thorbert-Mros et al., 2017). However, in a study of 25-year-old Norwegians, Åstrøm (2008) found that only 10.9% reported daily flossing, and a longitudinal study showed that when assessed at 15, 23 and 30 years of age, daily flossing was lowest among 23-year-olds (Åstrøm & Wold, 2012).

In order to better promote flossing among young adults, it is crucial to understand the barriers to flossing behaviour. One explanation that has received little attention concerns executive functioning (EF) capacity and the role this plays for behavioural self-regulation among young adults. EF refers to processes performed by the central executive, the component of working memory responsible for maintaining conscious attention to information (Miyake et al., 2000). From early childhood, gradual development of the central executive aligns strongly with the capacity to exercise top-down self-control over behaviour, until both EF and self-regulation capacity reach a degree of stability in early adulthood (Bridgett et al., 2015). In the context of flossing, self-regulation refers to the ability to monitor and initiate flossing behaviour. Even in adulthood, individuals may differ substantially regarding capacity for behavioural self-regulation (Miyake & Friedman, 2012), and these inter-personal differences can be attributed to EF and its role in facilitating the manipulation of cognitive information, focusing of attention, megacognitive reflection, and problem solving (Diamond, 2013; Hofmann et al., 2012).

EF and self-regulation processes also represent methods of engaging in conscious information processing, as opposed to allowing automated tendencies and impulses to dictate behaviour (Diamond, 2013). As impulses and tendencies can differ in their strength, EF processes are also differentiated along a continuum from ‘hot’ to ‘cold’, based on the extent to which they are associated with either raw information processing, or controlling real-time reactions to emotive or reward stimuli in the environment (Nejati et al., 2018; Salehinejad et al., 2021; Zelazo, 2020). In this sense, ‘colder’ functions represent more rational information processing in the absence of impulsive reactions (e.g. working memory, problem-solving, planning), while ‘hotter’ functions represent the processing of information that is attached to emotional, motivational or reward components (e.g. emotional control, delay of gratification) (Hofmann et al., 2012; Salehinejad et al., 2021). Applied to flossing, ‘cold’ functions are expected to be utilised when planning and visualising flossing behaviours, while ‘hot’ functions are expected to be involved in actively suppressing negative emotions or automated action tendencies that lead to flossing avoidance or the pursuit of alternative behaviours.

Within the health sector, exploration of the link between EF and behaviour has received considerable attention over the last decade. EF is recognised as essential in both inhibiting tendencies towards risk-behaviour, and overriding automated routines that interfere with adopting health-positive behaviours (Allan et al., 2016). Eating behaviour, for example, has been explored quite extensively and linked with several components of EF (Vainik et al., 2013), with interventions targeting EF showing promise in positively altering eating behaviour (Jones et al., 2018). Although the evidence is less conclusive regarding general health behaviours, a recent meta-analysis found a small overall effect of EF on both protective and risk-related health behaviour (Gray-Burrows et al., 2019). Flossing behaviour, however, has yet to be explored through the lens of EF.

### The current study

The present study investigated the role of EF on flossing behaviour and used Temporal Self-Regulation Theory (TST; Hall & Fong, 2007, 2013) to explore this association. TST suggests that the effect of pre-formed intentions on actual behaviour is moderated by two forces: the degree to which individuals can autonomously control their behaviour in a specific situation, i.e. EF-capacity, and the level of *behavioural prepotency* (BP), i.e. the degree to which the behaviour is automated or habitual and supported by the environment (Hall & Fong, 2007). While intention represents an explicit drive to perform a given behaviour, BP represents a more implicit measure of the extent to which the behaviour is already supported. Applications of TST have illustrated its ability to explain risk behaviours such as alcohol consumption (Black et al., 2017; Murray & Mullan, 2019), snacking behaviour (Elliston et al., 2017; Evans et al., 2017), and sweetened-beverage intake (McAlpine & Mullan, 2022; Moran & Mullan, 2021). Similarly, TST has been shown to explain variability in protective health behaviours, such as fruit and vegetable consumption (Evans et al., 2017; Frye & Shapiro, 2021), intake of vitamin supplements (Allom et al., 2018), physical activity (Frye & Shapiro, 2021), adherence to medication plans (Liddelow et al., 2021), and hand-hygiene practice (Liddelow et al., 2021). Recent interventions based on TST also showed benefits in improving self-care regimens among patients with heart disease (Chew et al., 2021). With no explicit applications of TST or EF to the study of flossing behaviours, the current study sought to bridge this gap. It was anticipated that the TST framework would be transferable to the modelling of flossing engagement. One concern, however, laid in the definition of EF that was to be applied. Along the continuum from ‘hot’ to ‘cold’ processes, EF refers to a number of sub-functions that are recognised to possess both unique and overlapping features (Miyake et al., 2000; Miyake & Friedman, 2012). With little established evidence pertaining to EF and flossing behaviour, determining how to measure EF in this context was not straight-forward. A secondary aim was therefore developed to investigate various sub-domains of EF as a means of highlighting those executive functions, or groups of functions, that best explain flossing behaviour. Exploring sub-domains was also performed with future applications in mind, where increased clarity and specificity regarding relevant EF-functions is anticipated to benefit intervention development (Michie et al., 2005). The Behaviour Rating Inventory of Executive Function – Adult Version (BRIEF-A; Roth et al., 2005) was judged to be a measure of EF that suited this purpose, providing insight into general EF and several sub-domains along the hot-cold continuum.

The current study thus aimed to apply TST to engagement in flossing behaviour using the BRIEF-A as a means of operationalising EF. The study compared different definitions of EF sub-domains with a goal to identify the functions that best explained flossing behaviour. It was expected that global EF would moderate the relationship between intentions and behaviour within the TST framework, and that the overall model would explain a significant amount of variability in flossing frequency. Concerning individual EF processes, although there was little available evidence to guide hypotheses, it was expected that self-monitoring would represent a fitting definition of EF within a TST framework based on a positive relationship between self-monitoring interventions and oral hygiene behaviours (Newton & Asimakopoulou, 2015; Rogers et al., 2022).

## Materials and methods

The study tested hypotheses following from the TST using a cross-sectional design and self-report questionnaire. Based on previous findings (Black et al., 2017; Evans et al., 2017; Moran & Mullan, 2021; Murray & Mullan, 2019), interaction effects between EF and health behaviour were typically small, approximately *f* ^2^ = 0*.*03. To determine the necessary sample-size, calculations were made using G*Power (Faul et al., 2007, 2009), with effect size set to *f* ^2^ = 0*.*03, power set to *β *= 0*.*80 and alpha-level set to *α *= 0*.*05 for a fixed multiple-regression model with five covariates (three direct effects and two interactions). The minimum sample size was *N *= 264.

All study materials were approved by the Faculty of Dentistry at the University of Oslo. The study was given exemption by the Regional Committee for Medical Research Ethics (ref: 262969), and data-privacy approved by the Norwegian Center for Research Data (ref: 102719). After obtaining informed consent, participants completed a digital survey delivered using the Nettskjema (https://nettskjema.no/) platform. Participants were recruited from universities within Norway using student-portals, newsletters, student-focused social-media pages, and physically through posters and brochures containing a QR-code. Age was restricted to 18–30 years, reflecting a young adult sample, and corresponding to the age-matched normative data of the BRIEF-A. An incentive for participation was being entered into a lottery to win one of ten gift cards worth NOK 500kr (approx. EUR €50).

### Measures

Prior to the study, a pilot questionnaire was completed by 28 Norwegian undergraduate students to assess the suitability of the intention, BP, and flossing measures, with positive results.

#### Intention

The present study measured explicit behavioural intention using three items: I (intend/plan/expect) to clean between my teeth at least once each day. These items demonstrated good reliability and utility during previous applications (Nystrand & Olsen, 2020; Rise & Ommundsen, 2011), and during the pilot study. Cronbach’s alpha in the current study was *α *= 0*.*93.

#### Behavioural prepotency

BP was derived from self-reported habit strength and environmental cue-exposure using confirmatory factor analysis via the Lavaan package for R (Rosseel, 2012) to produce a single-factor solution, mirroring methods used by Black et al. (2017). Combining habit-strength and environmental cue-exposure reflected the hypothesis derived from the TST that these constructs share commonalities related to the construct of implicit BP (Hall & Fong, 2013), which may differ from explicit intentions. The methods used to quantify habit strength and environmental cue-exposure were as follows:

*Habit strength*. The strength of flossing habits was measured using the Self-Report Behavioural Automaticity Index (Gardner et al., 2012), a reduced version of the Self-Report Habit Index (Verplanken & Orbell, 2003). The scale uses four items related to habit strength: Flossing at least once each day is something I (do automatically/do without having to consciously remember/do without thinking/start doing before I realise I’m doing it). Responses were recorded using 5-point Likert scales (strongly disagree – strongly agree). Pilot data demonstrated good reliability. Cronbach’s alpha in the present study was *α *= 0*.*96.

*Environmental cue exposure*. Exposure to environmental cues was assessed using a structured version of the Cues to Action Scale (CTAS; Booker & Mullan, 2013). While the original scale required participants to imagine and evaluate personally relevant cues, the present study mirrored methods used by Evans et al. (2017), with common cues (*k *= 4) elicited from pilot data, allowing cue exposure to be measured using these cues alone and 5-point Likert scales (never – several times per day). Care was taken that cues were representative of the original cue-categories proposed by Booker and Mullan (2013). The items were: How often do you notice a floss container (physical cue) in the bathroom? How often do you think about upcoming social events (social cues)? How often do you feel too exhausted (emotional cue) to care about your teeth? How often do you think about commitments (internal cues) to create positive health-habits? Root mean square error of approximation (RMSEA) was employed to determine whether the factor solution had sufficient fit to the data. Although initial testing revealed sub-optimal fit (*RMSEA *= 0*.*072), based on a cut-off of *RMSEA < *0*.*06 (Hu & Bentler, 1999), the exploration of modification indices attributed poor fit to CTAS items related to social and internal cues. Removing the CTAS item related to internal cues had the most positive impact on model fit (*RMSEA *= 0*.*051) with the resulting factor solution used to represent BP. Details of the factor analysis are included in Appendix B.

#### Executive functioning

EF was measured using the Behaviour Rating Inventory of Executive Function – Adult Version (Roth et al., 2005). The BRIEF-A is a 75-item self-report questionnaire evaluating engagement in everyday behaviours that are linked to specific domains of EF. Respondents indicate difficulties with such behaviours (rated as *often*, *sometimes*, or *never* being a problem) during the past four weeks, with results providing both a Global Executive Composite (GEC) of overall functioning, as well as scores on two primary indices composed of nine unique sub-scales. These sub-scales provide information about unique processes that range along the hot-cold continuum of EF (Zelazo, 2020). The first is the Metacognition Index (MI), comprised of five sub-scales related to neutral self-reflective thinking: Initiation (8 items), Working Memory (8 items), Planning/Organisation (10 items), Task Monitoring (6 items), and Organisation of Materials (8 items). The second, related to thinking in the presence of external stimuli or rewards, is the Behavioural Regulation Index (BRI), which consisted of four sub-scales: Inhibition (8 items), Shifting (6 items), Emotional Control (10 items), and Self-Monitoring (6 items). Higher scores on the BRIEF-A indicate more frequent difficulties with EF. As the BRIEF-A is standardised, scores can be interpreted relative to age-corrected normative data, and the instrument contains three separate measures of response validity to assess the negativity (tendency to answer negatively), infrequency (tendency to give unexpected answers), or inconsistency (tendency for answers to contradict) of participant responses. Participants were excluded if any raw scores on validity scales exceeded the recommended cut-off values: negativity >6 items, infrequency >3 items, and inconsistency >8 items (Roth et al., 2005) The psychometric validity of the BRIEF-A and its sub-scales were established during the development of the scale, and a Norwegian version of the scale has been used frequently among Norwegian samples (e.g. Grane et al., 2014; Løvstad et al., 2016, 2012). Cronbach’s alpha for GEC in the current study was *α *= 0*.*96. All indices and sub-scales also demonstrated high (*>* 0*.*70) consistency, with values included in Appendix A.

### Flossing frequency

Flossing frequency was assessed initially using three items that examined flossing at the daily, weekly, and monthly level. Looking at the distributions of participant responses, daily and weekly measures both exhibited bimodal distributions that gave little information about between-participant variation in routines and did not fit the assumptions of the planned analyses. Monthly flossing, however, exhibited a more even distribution and was therefore used as the dependent variable. Monthly flossing was measured using a single item ‘In the last month, approximately how often have you used floss?’, with responses given on a 5-point Likert scale (never, a few times per month, at least once a week, several times each week, every day).

### Analysis

A key hypothesis of TST is that BP and EF moderate the intention-behaviour relationship. Therefore, primary analyses concerned model fit (*R*^2^) and the magnitude of moderation effects. While BP was operationalised consistently across analyses, individual EF-processes were anticipated to have potentially independent effects on the outcome of interest; with a secondary aim being to contrast these effects. To achieve this, a series of multivariate linear regression models were constructed, moving down the hierarchy from global to specific EF-processes, testing the influence of different sub-domains of EF within the given additive multiple moderator model (Hayes, 2017; Montoya, 2019).

Analyses were performed using R-Studio (RStudio Team, 2020) and both the Lavaan package (Rosseel, 2012) and PROCESS macro (Hayes, 2017). As per Hayes and Rockwood (2017), independent variables were centred prior to the moderation analyses. Demographic covariates were excluded from the model as there was little theoretical evidence supporting their inclusion within this context. Rather, the chosen sampling design was anticipated to sufficiently control for potential confounders. To help visualise moderation effects related to EF, simple slopes were plotted while controlling for BP, further facilitating Johnson and Neyman (1938) testing to determine the regions of effect-significance. The alpha level for all significance tests was set to *α* ≤ 0.05.

## Ethics statement

Institutional Review Board Statement: The study was conducted in accordance with the Declaration of Helsinki and was approved by an Institutional Review Board/Ethics committee. See details under Methods. The study received an exemption from an Institutional Review Board/Ethics committee. See details under Methods.

## Results

A total of 383 participants completed the study. Five respondents were removed based on age and 16 based on the validity of responses to the BRIEF-A: 11 based on infrequency, 3 based on inconsistency, and 2 based on both negativity and inconsistency. Data were also screened for outliers based on interquartile range, however none were detected. After cleaning, data from the remaining 362 participants were used for the analysis. The age of the sample was *M *= 23*.*25 (*SD *= 3*.*12), with 76.5% female.

Descriptive statistics and correlations between key variables are presented in Table 1. An extended version of the table including all subscales of the BRIEF-A is provided in Appendix A. Note that higher scores on the BRIEF-A indicate difficulties in that domain of EF, meaning negative correlations between EF and flossing behaviour were anticipated.

**Table 1.** Means, standard deviations, ranges, and zero-order correlations between key variables.

Variable	Range	M (SD)	1	2	3	4	5
1. Intention	3 - 15	10.20 (4.66)					
2. BP	−0.92 - 1.70	0.00 (0.99)	0.71***				
3. BRIEF-A: BRI	30 - 74	44.93 (9.68)	−0.07*ns*	−0.14***			
4. BRIEF-A: MI	40 - 115	63.59 (13.72)	−0.12*	−0.21***	0.77***		
5. BRIEF-A: GEC	72 - 173	108.50 (22.06)	−0.10*	−0.19***	0.92***	0.96***	
6. Monthly flossing	1 - 5	3.16 (1.51)	0.76***	0.81***	−0.09*ns*	−0.13***	−0.12*

BP = Behavioural Prepotency, BRIEF-A: BRI = Behavioural Regulation Index, BRIEF-A: MI = Metacognition Index, BRIEF-A: GEC = Global Executive Composite, * = *p* < 0.05, ** = *p* < 0.01, *** = *p* < 0.001, ns = non-significant.

Hierarchical output from the multiple-moderator regression model predicting monthly flossing frequency, using GEC to represent EF-capacity, is presented in Table 2. At the first step, intention alone explained 57.85% of variance in flossing behaviour. At step two, BP and GEC helped explain an additional 14.90% of variance. At step three, interactions explained an additional 1.19% of variance, with total variance explained 73.94%. No direct effect for GEC was detected, and the moderation effect of GEC on the intention-behaviour relationship was small but significant (*β *= *−*0*.*06*, p *= 0*.*037). Of note were conditional tables provided by the PROCESS macro (Hayes, 2017), describing the direct effect of intention on flossing given various levels of BP and EF. Conditional tables showed that the effect of intention on behaviour was insignificant at elevated levels of BP, independent of EF. Conditional tables and full output are available in Appendix C.

**Table 2.** Hierarchical linear regression modelling self-reported monthly flossing frequency and using the global executive composite as a measure of general executive functioning.

	Variable	*β*	*SE*	*p*-value	*R*2	*R*^2^*t:,*	*F*
Step 1	Intention	0.76	0.01	<0.001***	0.58	0.58	494.0
Step 2	Intention	0.37	0.01	<0.001***	0.73	0.15	318.7
	BP	0.56	0.06	<0.001***			
	GEC	0.02	0.00	0.42*ns*			
Step 3	Intention	0.25	0.02	<0.001***	0.74	0.01	202.0
	BP	0.68	0.08	<0.001***			
	GEC	0.01	0.00	0.73*ns*			
	Intention x BP	−0.14	0.02	<0.001***			
	Intention x GEC	−0.06	0.00	0.04*			

BP = Behavioural Prepotency, GEC = Global Executive Composite, * = *p* < 0.05, ** = *p* < 0.01, *** = *p* < 0.001, ns = non-significant.

Going beyond the use of GEC to represent EF-capacity, EF was further differentiated using the BRI and MI of the BRIEF-A. As step one was the same for these models, hierarchical output from steps 2 and 3 are presented in Table 3. For the BRI model, interactions explained an additional 1.33% of variance and the moderation effect of BRI was significant (*β *= *−*0*.*07*, p *= 0*.*014). For the MI model, interactions explained an additional 1.05% of variance, but no direct or moderating effects were found. The BRI was deemed a better reflection of EF-capacity related to flossing behaviour and its sub-domains alone were explored further.

**Table 3.** Comparison of hierarchical linear regressions modelling self-reported monthly flossing frequency using either the BRIEF-a behavioural regulation index or metacognition index to measure executive functioning.

Variable		*β*	*SE*	*p*-value	*R*^2^	*R*^2^Δ	*F*
*Behavioural Regulation Index (BRI)*							
Step 2	Intention	0.37	0.01	<0.001***	0.73	0.15	318.2
	BP	0.55	0.06	<0.001***			
	BRI	0.01	0.00	0.62*ns*			
Step 3	Intention	0.25	0.02	<0.001***	0.74	0.01	203.2
	BP	0.69	0.08	<0.001***			
	BRI	0.00	0.00	0.93*ns*			
	Intention x BP	−0.14	0.02	<0.001***			
	Intention x BRI	−0.07	0.00	0.01*			
*Metacognition Index (MI)*							
Step 2	Intention	0.37	0.01	<0.001***	0.73	0.15	319.0
	BP	0.56	0.06	<0.001***			
	MI	0.03	0.00	0.34*ns*			
Step 3	Intention	0.25	0.02	<0.001***	0.74	0.01	200.8
	BP	0.68	0.08	<0.001***			
	MI	0.01	0.00	0.64*ns*			
	Intention x BP	−0.14	0.02	<0.001***			
	Intention x MI	−0.04	0.00	0.11*ns*			

BP = Behavioural Prepotency, BRI = Behavioural Regulation Index, MI = Metacognition Index, * = *p* < 0.05, ** = *p* < 0.01, *** = *p* < 0.001, ns = non-significant.

The four sub-scales of the BRI were entered into separate TST models. Output regarding the coefficients related to the direct and moderation effects, as well as model fit, is presented in Table 4. The output showed that specific functions related to shifting and emotional control best explained flossing behaviour.

**Table 4.** Direct (β_D_) and interaction (β_I_) coefficients of behavioural regulation index subscales from the behaviour rating inventory of executive function – adult version.

Variable	*β_D_*	*β_I_*	*R^2^_model_*
Inhibit	−0.01ns	−0.04ns	0.7375
Shift	0.01ns	−0.07*	0.7406
Emotional Control	0.01ns	−0.07**	0.7412
Self-Monitor	0.00ns	−0.04ns	0.7375

* = *p* < 0.05, ** = *p* < 0.01, *** = *p* < 0.001, ns = non-significant.

### Simple slopes

Plotting of simple slopes was performed to visualise relationships with specific EF processes and explore observations from the conditional tables. To achieve this, data were mean-split based on BP into high-BP (*n *= 142) and low-BP (*n *= 220) groups. The slopes represent the first and third quartile-values of the moderators. For ease of interpretation, EF labels are reversed so higher scores reflect positive EF-capacity, rather than EF-difficulties. All output used to generate plots is included in Appendix D.

**Figure 1.**
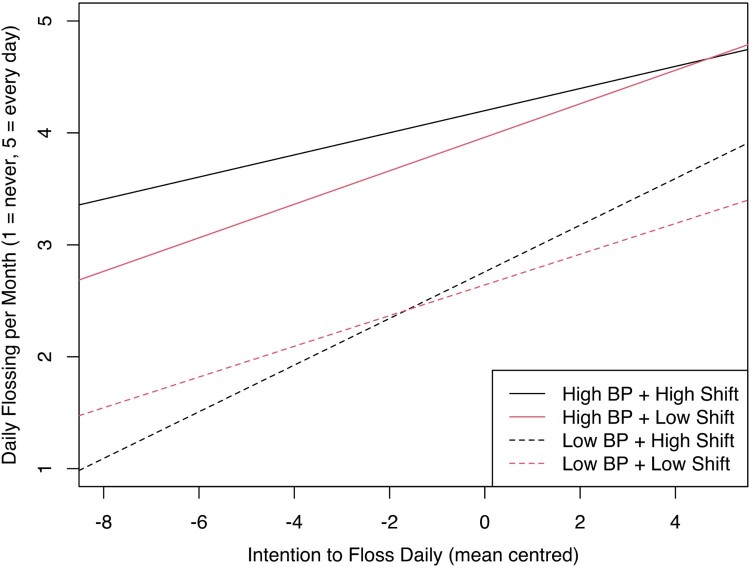
Effect of shifting (High Shift vs Low Shift) as a moderator of the relationship between intention and monthly flossing, with data split by behavioural prepotency (High BP vs Low BP).

The influence of shifting on the intention-behaviour relationship, relative to BP-level, is plotted in Figure 1. Analysis of the low-BP group revealed a significant moderation effect for shifting (*β *= *−*0*.*18*, p *= 0*.*009). Johnson-Neyman testing indicated that moderation effects were present for all levels of shifting except for the 2.3% of participants reporting the most shifting difficulties. Within the high-BP group, the moderation effect was not significant.

**Figure 2.**
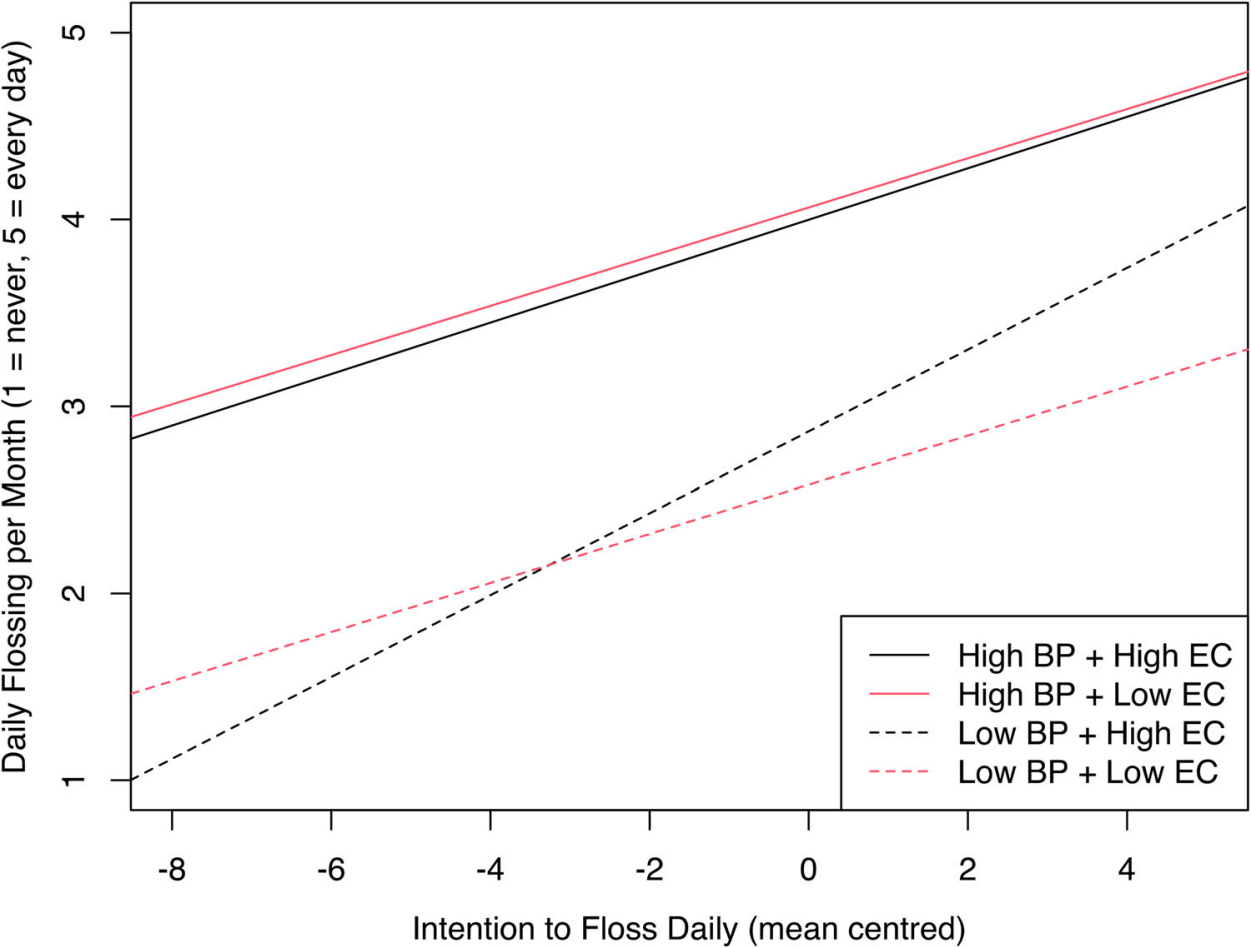
Effect of emotional control (High EC vs Low EC) as a moderator of the relationship between intention and monthly flossing, with data split by behavioural prepotency (High BP vs Low BP)*.*

The influence of emotional control on the intention-behaviour relationship, based on BP, is plotted in Figure 2. The influence of emotional control for those in the low-BP group again revealed a significant moderation effect (*β *= *−*0*.*27*, p < *0*.*001). Johnsen-Neyman testing indicated the moderation effect was present for all levels of emotional control except for the 6.8% of participants reporting the most emotional control difficulties. Again, within the high-BP group, moderation effects were not detected.

For both EF processes, plots indicated that the translation of intention to behaviour was largely facilitated by environmental support (i.e. BP), reflected by the vertical distance between solid and dotted lines. Similarly, the plots indicated that increased capacity for EF provided a positive effect when BP was low, with these effects dissipating among high-BP groups.

## Discussion

The present study applied TST to the investigation of flossing behaviour among young adults. In addition to testing hypotheses derived from TST, the study aimed to examine which components of EF best defined this construct within the model. The study found that for self-reported monthly flossing behaviour, hypotheses were supported: BP and EF both moderated the intention-behaviour relationship in the anticipated directions. Examining the sub-domains of EF, the study revealed that behavioural-regulation functions, leaning towards the ‘hot’ end of the continuum, exhibited a better fit than metacognitive functions within the present model, and that specific functions related to shifting and emotional control were significant moderators of flossing. Moderation relationships were probed further, finding that the effect of EF-capacity on flossing behaviour may be more relevant for those with lower levels of BP. Possible interpretations of these findings are discussed.

### Utility of temporal self-regulation theory

Before interpreting more exploratory findings, an important result was the utility of TST for explaining flossing behaviour among young adults. Overall, the suitability was unsurprising. TST has been shown to explain a variety of health-behaviours and engagement in healthy-lifestyle behaviour (Booker & Mullan, 2013). However, some irregularities between current results and the existing literature were noteworthy, namely the high amount of variance explained by the present model (*R*^2^ = 0*.*74).

To offer an explanation for this strength, it could be based on the operationalisation of BP. A large portion of variance explained was attributable to direct– and moderation-effects attached to BP. While behavioural automaticity has been shown to moderate the intention-behaviour relationship pertinent to flossing (Gardner et al., 2016), the magnitude of variance explained was still greater than expected and might be attributed to the factor analysis used to measure the construct itself. This methodology was borrowed from Black et al. (2017), who accounted for a large amount of variance (*R*^2^ = 0*.*61) in alcohol-related behaviour using factor analysis techniques to capture the collective *psychological inertia* of behaviour (Hall & Fong, 2013). In the present study, connectedness to external and internal environments was quantified by the CTAS and SRBAI, respectively.

Additionally, the standardised Cues to Action Scale represented a second method that may have improved results through focusing the BP instrument towards between-participant commonalities. This version was developed using pilot data to elucidate the environmental cues most relevant to the target population, with this methodology borrowed from Evans et al. (2017). Compared to the available literature, they also explained a high degree of variance (*R*^2^ = 0*.*64) in their target behaviour using TST. The results suggest that quantifying cue-exposure in this way is more likely to capture shared tendencies between participants, allowing for more comparable scores, and a better representation of cue-exposure relative to the given sample.

Overall, the study demonstrated that TST may be suitable for explaining flossing behaviour. Moreover, there may be considerable strengths in standardising the methods used to quantify environmental cues, and using factor analysis to operationalise BP.

### Executive functioning

The more novel focus of the present study was to explore the influence of EF on flossing behaviour. A small but significant correlation between GEC and flossing mirrored a recent meta-analysis that demonstrated similar small significant correlations between EF-capacity and health-protective behaviours (Gray-Burrows et al., 2019). Further, as anticipated, EF moderated flossing frequency. It should be noted that although the moderation effect of EF constitutes a key hypothesis following from TST (Hall & Fong, 2007, 2013), significant moderation effects have only been observed for health-related behaviours on limited occasions (Allom et al., 2018; Black et al., 2017; Hall et al., 2008). Findings may again be explained by methodological differences.

Specifically, discrepancies could be based on the definition of EF, with multiple domains proposed to exist (Miyake et al., 2000; Stuss, 2011). In their meta-analysis, Gray-Burrows et al. (2019) pointed to this diversity in definitions as a potential source of heterogeneity, with different types of behaviour relating to alternate operationalizations of EF. The present study and comparison of primary indices from the BRIEF-A illustrated these discrepancies, with significant moderation effects associated with the BRI, but not MI. These results indicate that in a hypothetical situation where the study only tested metacognitive aspects of EF capacity, the conclusions regarding EF would have been very different and potentially misguided.

### Executive functioning as a moderator

Shifting and emotional control were both identified as moderators of the intention-behaviour relationship within the TST model. Shifting, as an independent process, represents the capacity to move flexibly from one activity or situation to another. When one task has captured our attention, shifting refers to the refocusing of attention and transitioning towards a new activity while avoiding rumination about the previous task (Roth et al., 2005). Emotional control, as a separate process, refers to the ability of an individual to flexibly modulate their emotional reactions according to the current context. This may include reappraising and changing emotions as they arise, and suppressing unwanted reactions to emotional states (Gross & John, 2003).

#### Shifting

Current results indicated that the capacity to flexibly shift attentional focus moderated the association between intention and flossing behaviour at low levels of BP. Even as intentions increase, an individual with low shifting capacity and low environmental support may continue to encounter barriers focusing on the target behaviour in the presence of competing routines. Roth et al. (2005) suggested that for those experiencing shifting-difficulties, visible calendars and schedules may help aid the anticipation of routine changes, encouraging individuals to pre-empt and prepare for change well in advance.

#### Emotional control

Examining emotional control, links with flossing match general observations of a relationship between emotional control and health behaviour (Evers, 2017). Specifically, findings indicate that events producing emotional change prior to flossing, and the capacity to manage these stimuli, may influence subsequent flossing frequency. One explanation may be that, among participants, flossing represents a deviation from established routines. If this is the case, then individuals will favour routine-behaviour in such instances, bringing about negative emotional reactions despite positive intentions (Muraven & Baumeister, 2000). These negative reactions may implicate emotional control as necessary for successful self-regulation (Roth et al., 2005).

Negative reactions are also especially relevant for behaviours performed before sleep (e.g. flossing), when cognitive resources are likely to be more depleted. Deviation from routines at these times is anticipated to be met with greater resistance and increased vulnerability to negative emotions (Baumeister & Vohs, 2016). This hypothesis, that negative emotional reactions are related to flossing being non-habitual, conversely insinuates that emotional control capacity would become less relevant as flossing becomes more automated and less of a deviation from normal routines. Present findings supported this effect as well. The almost null effect of emotional control capacity among those with high BP suggests that emotional challenges may be less frequent when automation is established.

Roth et al. (2005) suggested that emotional control development may be initiated through measures as simple as discussing the presence of emotions prior to a planned behaviour. Increasing the awareness and acceptance of emotional states through discussion is suggested to represent a fundamental basis for improving emotional regulation (Teper et al., 2013). Beyond discussion, more specific aims might include increasing the capacity for *response modulation*, strategies focused on recognising and calming resulting negative emotional reactions (Gross, 2015). Examples of response modulation strategies may include counting backwards from ten, or taking ten deep breaths, before deciding whether to floss.

### Strengths and weaknesses

The current study suffered some limitations related to sample composition and self-reporting methodology. Sample composition revealed that 76.5% of respondents were female. The over-representation of females in a study of Norwegian university students, however, was not deemed unusual, based on previous studies of oral health among this demographic (79.4% female, Halvari et al., 2012), and national studies of Norwegian universities (Sivertsen et al., 2019; Sivertsen et al., 2022). Further, while the narrow sampling demographic of university students aged 18–30 years reduced potential confounders, the ability to draw more general conclusions may be limited and requires further investigation. Regarding the self-report measures, flossing-frequency over a 30-day period may have had limitations attached to recall accuracy. However, the monthly scale was used to capture actual behavioural variance and observe between-participant variations in behaviour that may be otherwise hidden across more limited timespans (Stull et al., 2009). Not only did the 30-day measure capture more variance than daily or weekly measures, but the distribution also matched better with the assumptions of the parametric linear regression techniques employed. Nevertheless, the use of objective or ambulatory measures to gather flossing data represents a standard that future research may explore. Further, the BRIEF-A is often used as a two-part measure. The second survey is completed by a party who has observed the target individual in their daily life, allowing for a complimentary report. The current study did not employ observer ratings. However, delivering the BRIEF-A as a stand-alone self-report measure is common (see Roth et al., 2013, for example, for a list of clinical and non-clinical applications), with this version being well validated (Roth et al., 2005), and applied to healthy young adult populations (e.g. Koven & Thomas, 2010; Rabin et al., 2011). In general, hypotheses generated by the current data may be strengthened using more objective measures of both behaviour and executive functioning, and studying non-student samples.

Evaluating the strengths of the present study, utilising the BRIEF-A in conjunction with TST provided considerable methodological advantages. The BRIEF-A represented a more reliable and specific instrument to assess EF and neuropsychological self-regulation capacity than those previously applied to flossing behaviour (see Rogers et al., 2022, for an overview). Similarly, TST provided a series of testable hypotheses addressing not only the relationship between EF and flossing, but also how EF-processes explain behavioural variability within a detailed framework accounting for theoretical covariates.

## Conclusion

The current study sought to both apply TST as a means of explaining flossing behaviour among young adults, and to explore the role of EF within the context of flossing. Overall, the study found that TST provided a suitable framework for modelling flossing behaviour, and that EF pertaining to behavioural regulation, specifically emotional control and shifting, tended to best explain observations. This information holds considerable importance for young adults who may find it difficult to embrace flossing-habits; a behaviour that will ultimately assist in the long-term prevention of dental disease. Rather than focussing solely on motivational approaches as a strategy for improving flossing regimes, the dental community may consider the importance of EF and BP, and targeting support towards these variables to facilitate behavioural change. As one of the first studies to examine flossing behaviour through the lens of EF and TST, future studies are encouraged to build upon the current findings; continuing to explore these theoretical relationships and the effect of interventions developed with the present findings in mind.

## Supplementary Material

Supplemental Material

## Data Availability

The data that support the findings of this study are available from the corresponding author upon reasonable request.
