# Supplementary material for: Executive functioning as a moderator of flossing behaviour among young adults: a temporal self-regulation theory perspective
Source: Health Psychol Behav Med. 2023 Aug 27;11(1):2249972. doi: 10.1080/21642850.2023.2249972 (PMC10461502; doi:10.1080/21642850.2023.2249972)
Supplement: Supplemental Material [file RHPB_A_2249972_SM3198.docx]

**Executive Functioning as a Moderator of Flossing Behaviour among Young Adults**

Appendix A - Descriptive Statistics

**Table 1.** Bivariate correlations between variables used in the current study, demographic variables, and all subscales and indices of the Behavioural Rating Inventory of Executive Function - Adult version.

|  | 1 | 2 | 3 | 4 | 5 | 6 | 7 | 8 | 9 | 10 | 11 | 12 | 13 | 14 | 15 | 16 | 17 |
| --- | --- | --- | --- | --- | --- | --- | --- | --- | --- | --- | --- | --- | --- | --- | --- | --- | --- |
| 1. Age | - |  |  |  |  |  |  |  |  |  |  |  |  |  |  |  |  |
| 2. Gender | 0.03 | - |  |  |  |  |  |  |  |  |  |  |  |  |  |  |  |
| 3. Intention | 0.02 | 0.09 | - |  |  |  |  |  |  |  |  |  |  |  |  |  |  |
| 4. Behavioural Prepotency | 0.03 | 0.01 | 0.71*** | - |  |  |  |  |  |  |  |  |  |  |  |  |  |
| 5. Inhibit | -0.09 | -0.07 | -0.08 | -0.12* | - |  |  |  |  |  |  |  |  |  |  |  |  |
| 6. Shift | 0.01 | 0.08 | -0.02 | -0.08 | 0.53^***^ | - |  |  |  |  |  |  |  |  |  |  |  |
| 7. Emotional Control | -0.01 | 0.23*** | -0.05 | -0.14** | 0.52^***^ | 0.56*** | - |  |  |  |  |  |  |  |  |  |  |
| 8. Self-Monitor | -0.09 | -0.06 | -0.08 | -0.11* | 0.69^***^ | 0.57*** | 0.55*** | - |  |  |  |  |  |  |  |  |  |
| 9. Behavioural Regulation Index | -0.05 | 0.09 | -0.07 | -0.14** | 0.81^***^ | 0.78*** | 0.86*** | 0.81*** | - |  |  |  |  |  |  |  |  |
| 10. Initiate | -0.00 | -0.06 | -0.08 | -0.16** | 0.58^***^ | 0.62*** | 0.42*** | 0.52*** | 0.63*** | - |  |  |  |  |  |  |  |
| 11. Working Memory | -0.05 | 0.02 | -0.10 | -0.19*** | 0.72^***^ | 0.61*** | 0.57*** | 0.62*** | 0.76*** | 0.71*** | - |  |  |  |  |  |  |
| 12. Plan/Organize | -0.01 | -0.02 | -0.13* | -0.17** | 0.69*** | 0.65*** | 0.51*** | 0.65*** | 0.74*** | 0.75*** | 0.75*** | - |  |  |  |  |  |
| 13. Task Monitor | -0.10* | -0.02 | -0.10 | -0.18*** | 0.65*** | 0.57*** | 0.46*** | 0.61*** | 0.67*** | 0.70*** | 0.75*** | 0.77*** | - |  |  |  |  |
| 14. Organization of Materials | 0.02 | 0.02 | -0.10 | -0.18*** | 0.50*** | 0.39*** | 0.30*** | 0.43*** | 0.48*** | 0.52*** | 0.54*** | 0.58*** | 0.56*** | - |  |  |  |
| 15. Metacognition Index | -0.03 | -0.01 | -0.12* | -0.21*** | 0.74*** | 0.67*** | 0.53*** | 0.66*** | 0.77*** | 0.86*** | 0.88*** | 0.91*** | 0.87*** | 0.75*** | - |  |  |
| 16. Global Executive Composite | -0.04 | 0.03 | -0.10* | -0.19*** | 0.82*** | 0.76*** | 0.71*** | 0.77*** | 0.92*** | 0.81*** | 0.88*** | 0.89*** | 0.84*** | 0.67*** | 0.96*** | - |  |
| 17. Monthly Flossing | 0.09 | 0.04 | 0.76*** | 0.81*** | -0.09 | -0.03 | -0.08 | -0.09 | -0.09 | -0.10 | -0.12* | -0.10 | -0.11* | -0.13* | -0.13* | -0.12* | - |

* = p<0.05, ** = p<0.01, *** = p<0.00

**Table 2.** Bivariate correlations between monthly flossing and the interaction terms associated with intention and both behavioural prepotency and the executive functions tested within the study.

|  | 1. | 2. |
| --- | --- | --- |
| 1. Monthly Flossing | - |  |
| 2. Intention x Behavioural Prepotency | 0.72*** | - |
| 3. Intention x Global Executive Composite | 0.64*** | 0.42*** |
| 4. Intention x Behavioural Regulation Index | 0.64*** | 0.43*** |
| 5. Intention x Metacognition Index | 0.63*** | 0.40*** |
| 6. Intention x Inhibit | 0.63*** | 0.44*** |
| 7. Intention x Shift | 0.61*** | 0.42*** |
| 8. Intention x Emotional Control | 0.57*** | 0.38*** |
| 9. Intention x Self-Monitor | 0.62*** | 0.43*** |

* = p<0.05, ** = p<0.01, *** = p<0.001

**Table 3.** Mean, standard deviation, range and Cronbach’s alpha of all variables used in the current study, including all subscales of the Behavioural Rating Inventory of Executive Function - Adult version.

|  | M | SD | Range | *α* |
| --- | --- | --- | --- | --- |
| Intention | 10.20 | 4.66 | 3.00 - 15.00 | 0.94 |
| Behavioural prepotency | 0.00 | 0.99 | -0.92 - 1.70 | 0.85 |
| Monthly flossing | 3.16 | 1.51 | 1.00 - 5.00 | - |
| Inhibition | 11.80 | 2.89 | 8.00 - 22.00 | 0.75 |
| Shifting | 9.38 | 2.47 | 6.00 - 18.00 | 0.76 |
| Emotional control | 15.27 | 4.28 | 10.00 - 29.00 | 0.88 |
| Self-monitoring | 8.47 | 2.11 | 6.00 - 14.00 | 0.73 |
| *Behavioural Regulation Index* | 44.93 | 9.68 | 30.00 - 74.00 | 0.92 |
| Initiate | 13.59 | 3.20 | 8.00 - 24.00 | 0.76 |
| Working memory | 12.61 | 3.47 | 8.00 - 24.00 | 0.84 |
| Plan/organize | 15.44 | 3.86 | 10.00 - 28.00 | 0.83 |
| Task monitor | 9.73 | 2.30 | 6.00 - 18.00 | 0.73 |
| Organization of materials | 12.22 | 3.21 | 8.00 - 24.00 | 0.82 |
| *Metacognition Index* | 63.59 | 13.72 | 40.00 - 115.00 | 0.94 |
| *General Executive Composite* | 108.50 | 22.06 | 72.00 - 173.00 | 0.96 |

Appendix B - Factor Analysis of Behavioural Prepotency

Model

bp_model <- '

# Define model

bp_fac =~ NA*srbai1 + srbai2 + srbai3 + srbai4 + ctas1 + ctas2 + ctas3 + ctas4 # Includes all CTAS variables

# Fixed factor variance bp_fac ~~ 1*bp_fac

'

bp_fit <- cfa(bp_model, data = data) data$bp <- predict(bp_fit)

summary(bp_fit, fit.measures=TRUE, standardized=TRUE, modindices = TRUE) lavaan 0.6-12 ended normally after 34 iterations

Estimator ML

Optimization method NLMINB

Number of model parameters 16

Number of observations 362

Model Test User Model:

| Test statistic | 62.494 |
| --- | --- |
| Degrees of freedom | 20 |
| P-value (Chi-square) | 0.000 |

Model Test Baseline Model:

| Test statistic | 2046.790 |
| --- | --- |
| Degrees of freedom | 28 |
| P-value | 0.000 |

User Model versus Baseline Model:

Comparative Fit Index (CFI) 0.979

Tucker-Lewis Index (TLI) 0.971

Loglikelihood and Information Criteria:

Loglikelihood user model (H0) -3789.590 Loglikelihood unrestricted model (H1) -3758.343

| Akaike (AIC) |  | 7611.180 |
| --- | --- | --- |
| Bayesian (BIC) |  | 7673.447 |
| Sample-size adjusted | Bayesian (BIC) | 7622.686 |

Root Mean Square Error of Approximation:

RMSEA 0.077 <-- This is much higher

than the desired 0.06 or lower

90 Percent confidence interval - lower 0.056

90 Percent confidence interval - upper 0.098

P-value RMSEA <= 0.05 0.020

Standardized Root Mean Square Residual:

SRMR 0.052

Parameter Estimates:

Standard errors Standard

Information Expected

Information saturated (h1) model Structured

| Latent Variables:  bp_fac =~ | Estimate | Std.Err | z-value | P(>\|z\|) | Std.lv | Std.all |
| --- | --- | --- | --- | --- | --- | --- |
| srbai1 | 1.550 | 0.065 | 23.859 | 0.000 | 1.550 | 0.940 |
| srbai2 | 1.564 | 0.061 | 25.636 | 0.000 | 1.564 | 0.977 |
| srbai3 | 1.505 | 0.061 | 24.508 | 0.000 | 1.505 | 0.954 |
| srbai4 | 1.174 | 0.059 | 19.977 | 0.000 | 1.174 | 0.846 |
| ctas1 | 0.616 | 0.069 | 8.974 | 0.000 | 0.616 | 0.452 |
| ctas2 | 0.011 | 0.045 | 0.254 | 0.799 | 0.011 | 0.014 |
| ctas3 | 0.266 | 0.052 | 5.084 | 0.000 | 0.266 | 0.265 |
| ctas4 | 0.048 | 0.051 | 0.950 | 0.342 | 0.048 | 0.051 |
| Variances: |  |  |  |  |  |  |
| Estimate  bp_fac 1.000 | | Std.Err | z-value | P(>\|z\|) | Std.lv 1.000 | Std.all 1.000 |
| .srbai1 | 0.315 | 0.029 | 10.723 | 0.000 | 0.315 | 0.116 |
| .srbai2 | 0.114 | 0.019 | 6.066 | 0.000 | 0.114 | 0.045 |
| .srbai3 | 0.222 | 0.023 | 9.625 | 0.000 | 0.222 | 0.089 |
| .srbai4 | 0.547 | 0.043 | 12.621 | 0.000 | 0.547 | 0.284 |
| .ctas1 | 1.480 | 0.111 | 13.372 | 0.000 | 1.480 | 0.796 |
| .ctas2 | 0.720 | 0.054 | 13.454 | 0.000 | 0.720 | 1.000 |
| .ctas3 | 0.932 | 0.069 | 13.429 | 0.000 | 0.932 | 0.930 |
| .ctas4 | 0.916 | 0.068 | 13.453 | 0.000 | 0.916 | 0.997 |

Modification Indices:

lhs op rhs mi epc sepc.lv sepc.all sepc.nox

1 srbai1 ~~ srbai2 5.511 0.072 0.072 0.382 0.382

2 srbai1 ~~ srbai3 2.744 -0.046 -0.046 -0.174 -0.174

3 srbai1 ~~ srbai4 4.212 -0.054 -0.054 -0.129 -0.129

4 srbai1 ~~ ctas1 10.048 0.127 0.127 0.185 0.185

5 srbai1 ~~ ctas2 1.531 -0.034 -0.034 -0.072 -0.072

6 srbai1 ~~ ctas3 3.193 0.056 0.056 0.104 0.104

7 srbai1 ~~ ctas4 0.311 -0.017 -0.017 -0.032 -0.032

8 srbai2 ~~ srbai3 0.888 -0.030 -0.030 -0.188 -0.188

9 srbai2 ~~ srbai4 0.404 -0.014 -0.014 -0.058 -0.058

10 srbai2 ~~ ctas1 2.534 -0.050 -0.050 -0.122 -0.122

11 srbai2 ~~ ctas2 0.003 -0.001 -0.001 -0.004 -0.004

12 srbai2 ~~ ctas3 0.760 -0.021 -0.021 -0.066 -0.066

13 srbai2 ~~ ctas4 0.001 0.001 0.001 0.003 0.003 14 srbai3 ~~ srbai4 8.578 0.069 0.069 0.198 0.198

15 srbai3 ~~ ctas1 0.234 -0.017 -0.017 -0.030 -0.030

16 srbai3 ~~ ctas2 0.327 0.014 0.014 0.035 0.035

17 srbai3 ~~ ctas3 0.010 0.003 0.003 0.006 0.006

18 srbai3 ~~ ctas4 0.008 -0.002 -0.002 -0.005 -0.005

19 srbai4 ~~ ctas1 1.137 -0.052 -0.052 -0.058 -0.058

20 srbai4 ~~ ctas2 0.154 0.013 0.013 0.021 0.021

21 srbai4 ~~ ctas3 1.408 -0.046 -0.046 -0.064 -0.064

22 srbai4 ~~ ctas4 0.020 0.005 0.005 0.008 0.008

23 ctas1 ~~ ctas2 2.083 0.079 0.079 0.076 0.076

24 ctas1 ~~ ctas3 0.002 -0.003 -0.003 -0.002 -0.002

25 ctas1 ~~ ctas4 11.480 0.208 0.208 0.179 0.179

26 ctas2 ~~ ctas3 0.229 0.021 0.021 0.025 0.025

27 ctas2 ~~ ctas4 19.289 0.187 0.187 0.231 0.231 <-- High MI for ctas2-ctas4 relationship

28 ctas3 ~~ ctas4 2.986 -0.084 -0.084 -0.091 -0.091

The high MI suggests that allowing ctas2 and ctas4 to covary, or removing one of these items, would improve model fit. As there is little prior data to warrant introducing a covariance relationship between two items, removing one represents a more logical path, if these changes lead to an improved RMSEA.

# Model 2 - Removing CTAS2 bp_model <- '

# Define model

bp_fac =~ NA*srbai1 + srbai2 + srbai3 + srbai4 + ctas1 + ctas3 + ctas4

# Fixed factor variance bp_fac ~~ 1*bp_fac

'

bp_fit <- cfa(bp_model, data = data) data$bp <- predict(bp_fit)

summary(bp_fit, fit.measures=TRUE, standardized=TRUE, modindices = TRUE) lavaan 0.6-12 ended normally after 23 iterations

Estimator ML

Optimization method NLMINB

Number of model parameters 14

Number of observations 362

Model Test User Model:

| Test statistic | 39.529 |
| --- | --- |
| Degrees of freedom | 14 |
| P-value (Chi-square) | 0.000 |

Model Test Baseline Model:

Test statistic 2023.761

Degrees of freedom 21

P-value 0.000

User Model versus Baseline Model:

Comparative Fit Index (CFI) 0.987

Tucker-Lewis Index (TLI) 0.981

Loglikelihood and Information Criteria:

| Loglikelihood user model (H0) | | | | -3335.395 | |  | |
| --- | --- | --- | --- | --- | --- | --- | --- |
| Loglikelihood unrestricted model (H1) | | | | -3315.630 | |  |  |
| Akaike (AIC) | | | | 6698.789 | |  |  |
| Bayesian (BIC) | | | | 6753.272 | |  |  |
| Sample-size adjusted Bayesian (BIC) | | | | 6708.857 | |  |  |
| Root Mean Square Error of Approximation: | | | |  | |  |  |
| RMSEA  but still above 0.06  90 Percent confidence interval - lower | | | | 0.071  0.045 | | <-- Lower than original, | |
| 90 Percent confidence interval - upper | | | | 0.098 | |  | |
| P-value RMSEA <= 0.05 | | | | 0.084 | |  | |
| Standardized Root Mean Square Residual: | | | |  | |  | |
| SRMR | | | | 0.037 | |  | |
| Parameter Estimates: | | | |  | |  | |
| Standard errors Information  Information saturated (h1) model | | | | Standard Expected Structured | |  | |
| Latent Variables: bp_fac =~ | Estimate | Std.Err | z-value | | P(>\|z\|) | Std.lv | Std.all |
| srbai1 | 1.550 | 0.065 | 23.859 | | 0.000 | 1.550 | 0.940 |
| srbai2 | 1.564 | 0.061 | 25.636 | | 0.000 | 1.564 | 0.977 |
| srbai3 | 1.505 | 0.061 | 24.507 | | 0.000 | 1.505 | 0.954 |
| srbai4 | 1.174 | 0.059 | 19.977 | | 0.000 | 1.174 | 0.846 |
| ctas1 | 0.616 | 0.069 | 8.973 | | 0.000 | 0.616 | 0.452 |
| ctas3 | 0.266 | 0.052 | 5.084 | | 0.000 | 0.266 | 0.265 |
| ctas4 | 0.048 | 0.051 | 0.949 | | 0.343 | 0.048 | 0.050 |
| Variances: |  |  |  |  |  |  |  |
| Estimate  bp_fac 1.000 | | Std.Err | z-value | | P(>\|z\|) | Std.lv 1.000 | Std.all 1.000 |
| .srbai1 | 0.315 | 0.029 | 10.722 | | 0.000 | 0.315 | 0.116 |
| .srbai2 | 0.114 | 0.019 | 6.066 | | 0.000 | 0.114 | 0.045 |
| .srbai3 | 0.222 | 0.023 | 9.626 | | 0.000 | 0.222 | 0.089 |
| .srbai4 | 0.547 | 0.043 | 12.621 | | 0.000 | 0.547 | 0.284 |
| .ctas1 | 1.480 | 0.111 | 13.372 | | 0.000 | 1.480 | 0.796 |
| .ctas3 | 0.932 | 0.069 | 13.429 | | 0.000 | 0.932 | 0.930 |

.ctas4 0.916 0.068 13.453 0.000 0.916 0.997

Modification Indices:

lhs op rhs mi epc sepc.lv sepc.all sepc.nox

1 srbai1 ~~ srbai2 5.472 0.072 0.072 0.380 0.380

2 srbai1 ~~ srbai3 2.749 -0.046 -0.046 -0.175 -0.175

3 srbai1 ~~ srbai4 4.215 -0.054 -0.054 -0.129 -0.129

4 srbai1 ~~ ctas1 10.057 0.127 0.127 0.186 0.186

5 srbai1 ~~ ctas3 3.195 0.056 0.056 0.104 0.104

6 srbai1 ~~ ctas4 0.306 -0.017 -0.017 -0.032 -0.032

7 srbai2 ~~ srbai3 0.878 -0.030 -0.030 -0.187 -0.187

8 srbai2 ~~ srbai4 0.403 -0.014 -0.014 -0.058 -0.058

9 srbai2 ~~ ctas1 2.525 -0.050 -0.050 -0.121 -0.121

10 srbai2 ~~ ctas3 0.758 -0.021 -0.021 -0.066 -0.066

11 srbai2 ~~ ctas4 0.002 0.001 0.001 0.004 0.004

12 srbai3 ~~ srbai4 8.585 0.069 0.069 0.198 0.198

13 srbai3 ~~ ctas1 0.232 -0.017 -0.017 -0.029 -0.029

14 srbai3 ~~ ctas3 0.011 0.003 0.003 0.006 0.006

15 srbai3 ~~ ctas4 0.007 -0.002 -0.002 -0.005 -0.005

16 srbai4 ~~ ctas1 1.135 -0.052 -0.052 -0.058 -0.058

17 srbai4 ~~ ctas3 1.407 -0.046 -0.046 -0.064 -0.064

18 srbai4 ~~ ctas4 0.021 0.006 0.006 0.008 0.008

19 ctas1 ~~ ctas3 0.002 -0.002 -0.002 -0.002 -0.002

20 ctas1 ~~ ctas4 11.485 0.208 0.208 0.179 0.179

21 ctas3 ~~ ctas4 2.985 -0.084 -0.084 -0.091 -0.091

# Model 3 - Removing CTAS4 bp_model <- '

# Define model

bp_fac =~ NA*srbai1 + srbai2 + srbai3 + srbai4 + ctas1 + ctas2 + ctas3

# Fixed factor variance bp_fac ~~ 1*bp_fac

'

bp_fit <- cfa(bp_model, data = data) data$bp <- predict(bp_fit)

summary(bp_fit, fit.measures=TRUE, standardized=TRUE, modindices = TRUE) lavaan 0.6-12 ended normally after 26 iterations

Estimator ML

Optimization method NLMINB

Number of model parameters 14

Number of observations 362

Model Test User Model:

| Test statistic | 28.625 |
| --- | --- |
| Degrees of freedom | 14 |
| P-value (Chi-square) | 0.012 |

Model Test Baseline Model:

| Test statistic | 2012.018 |
| --- | --- |
| Degrees of freedom | 21 |
| P-value | 0.000 |

User Model versus Baseline Model:

Comparative Fit Index (CFI) 0.993

Tucker-Lewis Index (TLI) 0.989

Loglikelihood and Information Criteria:

Loglikelihood user model (H0) -3291.862 Loglikelihood unrestricted model (H1) -3277.549

| Akaike (AIC) |  | 6611.724 |
| --- | --- | --- |
| Bayesian (BIC) |  | 6666.207 |
| Sample-size adjusted | Bayesian (BIC) | 6621.791 |

Root Mean Square Error of Approximation:

RMSEA 0.054 <- RMSEA below 0.06,

indicating good fit

90 Percent confidence interval - lower 0.025

90 Percent confidence interval - upper 0.082

P-value RMSEA <= 0.05 0.377

Standardized Root Mean Square Residual:

SRMR 0.020

Parameter Estimates:

Standard errors Standard

Information Expected

Information saturated (h1) model Structured

| Latent Variables:  bp_fac =~ | Estimate | Std.Err | z-value | P(>\|z\|) | Std.lv | Std.all |
| --- | --- | --- | --- | --- | --- | --- |
| srbai1 | 1.550 | 0.065 | 23.860 | 0.000 | 1.550 | 0.940 |
| srbai2 | 1.564 | 0.061 | 25.635 | 0.000 | 1.564 | 0.977 |
| srbai3 | 1.505 | 0.061 | 24.508 | 0.000 | 1.505 | 0.954 |
| srbai4 | 1.174 | 0.059 | 19.977 | 0.000 | 1.174 | 0.846 |
| ctas1 | 0.616 | 0.069 | 8.970 | 0.000 | 0.616 | 0.451 |
| ctas2 | 0.011 | 0.045 | 0.249 | 0.803 | 0.011 | 0.013 |
| ctas3 | 0.266 | 0.052 | 5.086 | 0.000 | 0.266 | 0.266 |
| Variances: | Estimate | Std.Err | z-value | P(>\|z\|) | Std.lv | Std.all |
| bp_fac | 1.000 |  |  |  | 1.000 | 1.000 |
| .srbai1 | 0.315 | 0.029 | 10.722 | 0.000 | 0.315 | 0.116 |
| .srbai2 | 0.114 | 0.019 | 6.067 | 0.000 | 0.114 | 0.045 |
| .srbai3 | 0.222 | 0.023 | 9.625 | 0.000 | 0.222 | 0.089 |
| .srbai4 | 0.547 | 0.043 | 12.621 | 0.000 | 0.547 | 0.284 |
| .ctas1 | 1.480 | 0.111 | 13.372 | 0.000 | 1.480 | 0.796 |
| .ctas2 | 0.720 | 0.054 | 13.454 | 0.000 | 0.720 | 1.000 |
| .ctas3 | 0.932 | 0.069 | 13.429 | 0.000 | 0.932 | 0.929 |

Modification Indicies:

|  | lhs | op | rhs | mi | epc | sepc.lv | sepc.all | sepc.nox |
| --- | --- | --- | --- | --- | --- | --- | --- | --- |
| 1 | srbai1 | ~~ | srbai2 | 5.467 | 0.072 | 0.072 | 0.380 | 0.380 |
| 2 | srbai1 | ~~ | srbai3 | 2.770 | -0.046 | -0.046 | -0.175 | -0.175 |
| 3 | srbai1 | ~~ | srbai4 | 4.218 | -0.054 | -0.054 | -0.129 | -0.129 |
| 4 | srbai1 | ~~ | ctas1 | 10.125 | 0.127 | 0.127 | 0.186 | 0.186 |
| 5 | srbai1 | ~~ | ctas2 | 1.491 | -0.034 | -0.034 | -0.071 | -0.071 |
| 6 | srbai1 | ~~ | ctas3 | 3.171 | 0.056 | 0.056 | 0.104 | 0.104 |
| 7 | srbai2 | ~~ | srbai3 | 0.887 | -0.030 | -0.030 | -0.188 | -0.188 |
| 8 | srbai2 | ~~ | srbai4 | 0.401 | -0.014 | -0.014 | -0.057 | -0.057 |
| 9 | srbai2 | ~~ | ctas1 | 2.447 | -0.049 | -0.049 | -0.119 | -0.119 |
| 10 | srbai2 | ~~ | ctas2 | 0.000 | 0.000 | 0.000 | -0.002 | -0.002 |
| 11 | srbai2 | ~~ | ctas3 | 0.784 | -0.022 | -0.022 | -0.067 | -0.067 |
| 12 | srbai3 | ~~ | srbai4 | 8.581 | 0.069 | 0.069 | 0.198 | 0.198 |
| 13 | srbai3 | ~~ | ctas1 | 0.220 | -0.016 | -0.016 | -0.029 | -0.029 |
| 14 | srbai3 | ~~ | ctas2 | 0.350 | 0.014 | 0.014 | 0.036 | 0.036 |
| 15 | srbai3 | ~~ | ctas3 | 0.009 | 0.003 | 0.003 | 0.006 | 0.006 |
| 16 | srbai4 | ~~ | ctas1 | 1.122 | -0.052 | -0.052 | -0.058 | -0.058 |
| 17 | srbai4 | ~~ | ctas2 | 0.161 | 0.014 | 0.014 | 0.022 | 0.022 |
| 18 | srbai4 | ~~ | ctas3 | 1.416 | -0.046 | -0.046 | -0.065 | -0.065 |
| 19 | ctas1 | ~~ | ctas2 | 2.090 | 0.079 | 0.079 | 0.076 | 0.076 |
| 20 | ctas1 | ~~ | ctas3 | 0.002 | -0.003 | -0.003 | -0.002 | -0.002 |
| 21 | ctas2 | ~~ | ctas3 | 0.230 | 0.021 | 0.021 | 0.025 | 0.025 |

# It can be concluded that removing CTAS4 represents the most logical method of improving the fit of the behavioural prepotency factor solution.

Appendix C: Output for separate models using different operationalizations of executive functioning

Appendix C - 1: Model using Global Executive Composite (GEC)

- process (data, y = "floss", x = "intention", w ="bp", z = "gec", model = 2, jn = 1)

********************* PROCESS for R Version 4.0.1 ********************* Written by Andrew F. Hayes, Ph.D. [www.afhayes.com](http://www.afhayes.com/)

Documentation available in Hayes (2022). [www.guilford.com/p/hayes3](http://www.guilford.com/p/hayes3)

*********************************************************************** Model : 2

Y : floss

X : intention W : bp

Z : gec Sample size: 362

*********************************************************************** Outcome Variable: floss

Model Summary:

| R  0.8599 | R-sq  0.7394 | MSE  0.6046 | F  202.0280 | df1  5.0000 | df2  356.0000 | p  0.0000 |
| --- | --- | --- | --- | --- | --- | --- |
| Model: | coeff | se | t | p | LLCI | ULCI |
| constant | 3.3516 | 0.0691 | 48.4763 | 0.0000 | 3.2156 | 3.4875 |
| intention | 0.0815 | 0.0164 | 4.9715 | 0.0000 | 0.0493 | 0.1137 |
| bp | 1.0443 | 0.0831 | 12.5745 | 0.0000 | 0.8810 | 1.2077 |
| Int_1 | -0.0619 | 0.0171 | -3.6239 | 0.0003 | -0.0954 | -0.0283 |
| gec | 0.0007 | 0.0019 | 0.3480 | 0.7281 | -0.0031 | 0.0044 |
| Int_2 | -0.0008 | 0.0004 | -2.0645 | 0.0397 | -0.0016 | -0.0000 |

Product terms key:

Int_1 : intention x bp Int_2 : intention x gec

Test(s) of highest order unconditional interaction(s):

|  | R2-chng | F | df1 | df2 | p |
| --- | --- | --- | --- | --- | --- |
| X*W | 0.0096 | 13.1326 | 1.0000 | 356.0000 | 0.0003 |
| X*Z | 0.0031 | 4.2622 | 1.0000 | 356.0000 | 0.0397 |
| BOTH | 0.0119 | 8.1073 | 2.0000 | 356.0000 | 0.0004 |

Focal predictor: intention (X) Moderator: bp (W) Moderator: gec (Z)

Conditional effects of the focal predictor at values of the moderator(s):

| bp | | gec | | effect | | se | | t | | p | | LLCI | | ULCI | |
| --- | --- | --- | --- | --- | --- | --- | --- | --- | --- | --- | --- | --- | --- | --- | --- |
| -0.8953 | | -22.5221 | | 0.1553 | | 0.0165 | | 9.4084 | | 0.0000 | | 0.1228 | | 0.1878 | |
| -0.8953 | | -3.5221 | | 0.1398 | | 0.0136 | | 10.3018 | | 0.0000 | | 0.1131 | | 0.1664 | |
| -0.8953 | | 23.4779 | | 0.1177 | | 0.0159 | | 7.3968 | | 0.0000 | | 0.0864 | | 0.1490 | |
| -0.4032 | | -22.5221 | | 0.1248 | | 0.0158 | | 7.8925 | | 0.0000 | | 0.0937 | | 0.1560 | |
| -0.4032 | | -3.5221 | | 0.1093 | | 0.0131 | | 8.3147 | | 0.0000 | | 0.0835 | | 0.1352 | |
| -0.4032 | | 23.4779 | | 0.0872 | | 0.0160 | | 5.4360 | | 0.0000 | | 0.0557 | | 0.1188 | |
| 1.5469 | | -22.5221 | | 0.0042 | | 0.0394 | | 0.1065 | | 0.9153 | | -0.0732 | | 0.0816 | |
| 1.5469 | | -3.5221 | | -0.0113 | | 0.0389 | | -0.2911 | | 0.7711 | | -0.0879 | | 0.0653 | |
| 1.5469 | | 23.4779 | | -0.0334 | | 0.0408 | | -0.8191 | | 0.4133 | | -0.1136 | | 0.0468 | |

******************** ANALYSIS NOTES AND ERRORS ************************ Level of confidence for all confidence intervals in output: 95

W values in conditional tables are the 16th, 50th, and 84th percentiles. Z values in conditional tables are the 16th, 50th, and 84th percentiles.

- data$int_bp <- data$intention * data$bp
- data$int_gec <- data$intention * data$gec
- tst_model <- 'floss ~ intention+bp+gec+int_bp+int_gec'
- tst_fit <- sem(tst_model, data = data)
- summary(tst_fit, fit.measures=TRUE, standardized=TRUE) lavaan 0.6-12 ended normally after 1 iterations

Estimator ML

Optimization method NLMINB

Number of model parameters 6

Number of observations 362

Model Test User Model:

Test statistic 0.000

Degrees of freedom 0

Model Test Baseline Model:

| Test statistic | 486.823 |
| --- | --- |
| Degrees of freedom | 5 |
| P-value | 0.000 |
| User Model versus Baseline Model: |  |
| Comparative Fit Index (CFI) | 1.000 |
| Tucker-Lewis Index (TLI) | 1.000 |
| Loglikelihood and Information Criteria: |  |
| Loglikelihood user model (H0) | -419.566 |
| Loglikelihood unrestricted model (H1) | -419.566 |
| Akaike (AIC) | 851.132 |

Bayesian (BIC) 874.482

Sample-size adjusted Bayesian (BIC) 855.447

Root Mean Square Error of Approximation:

RMSEA 0.000

90 Percent confidence interval - lower 0.000

90 Percent confidence interval - upper 0.000

P-value RMSEA <= 0.05 NA

Standardized Root Mean Square Residual:

SRMR 0.000

Parameter Estimates:

Standard errors Standard

Information Expected

Information saturated (h1) model Structured

| Regressions:  floss ~ | Estimate | Std.Err | z-value | P(>\|z\|) | Std.lv | Std.all |
| --- | --- | --- | --- | --- | --- | --- |
| intention | 0.081 | 0.016 | 5.013 | 0.000 | 0.081 | 0.251 |
| bp | 1.044 | 0.082 | 12.680 | 0.000 | 1.044 | 0.683 |
| gec | 0.001 | 0.002 | 0.351 | 0.726 | 0.001 | 0.010 |
| int_bp | -0.062 | 0.017 | -3.654 | 0.000 | -0.062 | -0.140 |
| int_gec | -0.001 | 0.000 | -2.082 | 0.037 | -0.001 | -0.057 |
| Variances: | Estimate | Std.Err | z-value | P(>\|z\|) | Std.lv | Std.all |
| .floss | 0.595 | 0.044 | 13.454 | 0.000 | 0.595 | 0.261 |

Appendix C - 2: Model using Behavioural Regulation Index (BRI)

- process (data, y = "floss", x = "intention", w ="bp", z = "bri", model = 2, jn = 1)

********************* PROCESS for R Version 4.0.1 ********************* Written by Andrew F. Hayes, Ph.D. [www.afhayes.com](http://www.afhayes.com/)

Documentation available in Hayes (2022). [www.guilford.com/p/hayes3](http://www.guilford.com/p/hayes3)

*********************************************************************** Model : 2

Y : floss

X : intention W : bp

Z : bri Sample size: 362

*********************************************************************** Outcome Variable: floss

Model Summary:

| R  0.8605 | R-sq  0.7405 | MSE  0.6021 | F  203.1917 | df1  5.0000 | df2  356.0000 | p  0.0000 |
| --- | --- | --- | --- | --- | --- | --- |
| Model: | coeff | se | t | p | LLCI | ULCI |
| constant | 3.3553 | 0.0689 | 48.7078 | 0.0000 | 3.2198 | 3.4907 |
| intention | 0.0806 | 0.0164 | 4.9264 | 0.0000 | 0.0484 | 0.1128 |
| bp | 1.0470 | 0.0828 | 12.6397 | 0.0000 | 0.8841 | 1.2099 |
| Int_1 | -0.0623 | 0.0170 | -3.6731 | 0.0003 | -0.0957 | -0.0290 |
| bri | 0.0004 | 0.0043 | 0.0849 | 0.9324 | -0.0081 | 0.0088 |
| Int_2 | -0.0021 | 0.0009 | -2.4391 | 0.0152 | -0.0039 | -0.0004 |

Product terms key:

Int_1 : intention x bp Int_2 : intention x bri

Test(s) of highest order unconditional interaction(s):

|  | R2-chng | F | df1 | df2 | p |
| --- | --- | --- | --- | --- | --- |
| X*W | 0.0098 | 13.4913 | 1.0000 | 356.0000 | 0.0003 |
| X*Z | 0.0043 | 5.9492 | 1.0000 | 356.0000 | 0.0152 |
| BOTH | 0.0133 | 9.1116 | 2.0000 | 356.0000 | 0.0001 |

Focal predictor: intention (X) Moderator: bp (W) Moderator: bri (Z)

Conditional effects of the focal predictor at values of the moderator(s):

| bp | bri | effect | se | t | p | LLCI | ULCI |
| --- | --- | --- | --- | --- | --- | --- | --- |
| -0.8953 | -8.9282 | 0.1555 | 0.0156 | 9.9457 | 0.0000 | 0.1247 | 0.1862 |

| -0.8953 | -1.9282 | 0.1405 | 0.0135 | 10.4025 | 0.0000 | 0.1140 | 0.1671 |
| --- | --- | --- | --- | --- | --- | --- | --- |
| -0.8953 | 10.0718 | 0.1149 | 0.0158 | 7.2561 | 0.0000 | 0.0838 | 0.1461 |
| -0.4032 | -8.9282 | 0.1248 | 0.0150 | 8.2912 | 0.0000 | 0.0952 | 0.1544 |
| -0.4032 | -1.9282 | 0.1098 | 0.0131 | 8.3717 | 0.0000 | 0.0840 | 0.1356 |
| -0.4032 | 10.0718 | 0.0842 | 0.0159 | 5.2924 | 0.0000 | 0.0529 | 0.1156 |
| 1.5469 | -8.9282 | 0.0032 | 0.0391 | 0.0817 | 0.9349 | -0.0737 | 0.0801 |
| 1.5469 | -1.9282 | -0.0117 | 0.0388 | -0.3027 | 0.7623 | -0.0880 | 0.0645 |
| 1.5469 | 10.0718 | -0.0373 | 0.0404 | -0.9231 | 0.3566 | -0.1169 | 0.0422 |

******************** ANALYSIS NOTES AND ERRORS ************************ Level of confidence for all confidence intervals in output: 95

W values in conditional tables are the 16th, 50th, and 84th percentiles. Z values in conditional tables are the 16th, 50th, and 84th percentiles.

- data$int_bp <- data$intention * data$bp
- data$int_bri <- data$intention * data$bri
- tst_model <- 'floss ~ intention+bp+bri+int_bp+int_bri'
- tst_fit <- sem(tst_model, data = data)
- summary(tst_fit, fit.measures=TRUE, standardized=TRUE) lavaan 0.6-12 ended normally after 1 iterations

Estimator ML

Optimization method NLMINB

Number of model parameters 6

Number of observations 362

Model Test User Model:

Test statistic 0.000

Degrees of freedom 0

Model Test Baseline Model:

| Test statistic | 488.361 |
| --- | --- |
| Degrees of freedom | 5 |
| P-value | 0.000 |
| User Model versus Baseline Model: |  |
| Comparative Fit Index (CFI) | 1.000 |
| Tucker-Lewis Index (TLI) | 1.000 |
| Loglikelihood and Information Criteria: |  |
| Loglikelihood user model (H0) | -418.797 |
| Loglikelihood unrestricted model (H1) | -418.797 |
| Akaike (AIC) | 849.593 |

Bayesian (BIC) 872.943

Sample-size adjusted Bayesian (BIC) 853.908

Root Mean Square Error of Approximation:

RMSEA 0.000

90 Percent confidence interval - lower 0.000

90 Percent confidence interval - upper 0.000

P-value RMSEA <= 0.05 NA

Standardized Root Mean Square Residual:

SRMR 0.000

Parameter Estimates:

Standard errors Standard

Information Expected

Information saturated (h1) model Structured

| Regressions:  floss ~ | Estimate | Std.Err | z-value | P(>\|z\|) | Std.lv | Std.all |
| --- | --- | --- | --- | --- | --- | --- |
| intention | 0.081 | 0.016 | 4.968 | 0.000 | 0.081 | 0.248 |
| bp | 1.047 | 0.082 | 12.746 | 0.000 | 1.047 | 0.685 |
| bri | 0.000 | 0.004 | 0.086 | 0.932 | 0.000 | 0.002 |
| int_bp | -0.062 | 0.017 | -3.704 | 0.000 | -0.062 | -0.142 |
| int_bri | -0.002 | 0.001 | -2.460 | 0.014 | -0.002 | -0.066 |
| Variances: |  |  |  |  |  |  |
| Estimate | | Std.Err | z-value | P(>\|z\|) | Std.lv | Std.all |
| .floss 0.592 | | 0.044 | 13.454 | 0.000 | 0.592 | 0.259 |

Appendix C - 3: Model using Metacognition Index (MI)

- process (data, y = "floss", x = "intention", w ="bp", z = "mi", model = 2, jn = 1)

********************* PROCESS for R Version 4.0.1 ********************* Written by Andrew F. Hayes, Ph.D. [www.afhayes.com](http://www.afhayes.com/)

Documentation available in Hayes (2022). [www.guilford.com/p/hayes3](http://www.guilford.com/p/hayes3)

*********************************************************************** Model : 2

Y : floss

X : intention W : bp

Z : mi Sample size: 362

*********************************************************************** Outcome Variable: floss

Model Summary:

| R  0.8592 | R-sq  0.7382 | MSE  0.6074 | F  200.7717 | df1  5.0000 | df2  356.0000 | p  0.0000 |
| --- | --- | --- | --- | --- | --- | --- |
| Model: | coeff | se | t | p | LLCI | ULCI |
| constant | 3.3495 | 0.0693 | 48.3016 | 0.0000 | 3.2131 | 3.4859 |
| intention | 0.0822 | 0.0164 | 5.0062 | 0.0000 | 0.0499 | 0.1146 |
| bp | 1.0437 | 0.0833 | 12.5322 | 0.0000 | 0.8799 | 1.2074 |
| Int_1 | -0.0610 | 0.0171 | -3.5576 | 0.0004 | -0.0947 | -0.0273 |
| mi | 0.0014 | 0.0031 | 0.4615 | 0.6447 | -0.0046 | 0.0075 |
| Int_2 | -0.0010 | 0.0007 | -1.5790 | 0.1152 | -0.0023 | 0.0003 |

Product terms key:

Int_1 : intention x bp Int_2 : intention x mi

Test(s) of highest order unconditional interaction(s):

|  | R2-chng | F | df1 | df2 | p |
| --- | --- | --- | --- | --- | --- |
| X*W | 0.0093 | 12.6565 | 1.0000 | 356.0000 | 0.0004 |
| X*Z | 0.0018 | 2.4932 | 1.0000 | 356.0000 | 0.1152 |
| BOTH | 0.0105 | 7.1228 | 2.0000 | 356.0000 | 0.0009 |

Focal predictor: intention (X) Moderator: bp (W) Moderator: mi (Z)

Conditional effects of the focal predictor at values of the moderator(s):

| bp | mi | effect | se | t | p | LLCI | ULCI |
| --- | --- | --- | --- | --- | --- | --- | --- |
| -0.8953 | -13.5939 | 0.1509 | 0.0167 | 9.0487 | 0.0000 | 0.1181 | 0.1837 |

| -0.8953 | -2.5939 | 0.1395 | 0.0137 | 10.1856 | 0.0000 | 0.1126 | 0.1664 |
| --- | --- | --- | --- | --- | --- | --- | --- |
| -0.8953 | 14.4061 | 0.1219 | 0.0159 | 7.6848 | 0.0000 | 0.0907 | 0.1531 |
| -0.4032 | -13.5939 | 0.1209 | 0.0160 | 7.5736 | 0.0000 | 0.0895 | 0.1523 |
| -0.4032 | -2.5939 | 0.1095 | 0.0132 | 8.2695 | 0.0000 | 0.0835 | 0.1356 |
| -0.4032 | 14.4061 | 0.0919 | 0.0160 | 5.7394 | 0.0000 | 0.0604 | 0.1234 |
| 1.5469 | -13.5939 | 0.0020 | 0.0395 | 0.0502 | 0.9600 | -0.0757 | 0.0797 |
| 1.5469 | -2.5939 | -0.0094 | 0.0391 | -0.2403 | 0.8102 | -0.0862 | 0.0674 |
| 1.5469 | 14.4061 | -0.0270 | 0.0409 | -0.6587 | 0.5105 | -0.1074 | 0.0535 |

******************** ANALYSIS NOTES AND ERRORS ************************ Level of confidence for all confidence intervals in output: 95

W values in conditional tables are the 16th, 50th, and 84th percentiles. Z values in conditional tables are the 16th, 50th, and 84th percentiles.

- data$int_bp <- data$intention * data$bp
- data$int_mi <- data$intention * data$mi
- tst_model <- 'floss ~ intention+bp+mi+int_bp+int_mi'
- tst_fit <- sem(tst_model, data = data)
- summary(tst_fit, fit.measures=TRUE, standardized=TRUE) lavaan 0.6-12 ended normally after 1 iterations

Estimator ML

Optimization method NLMINB

Number of model parameters 6

Number of observations 362

Model Test User Model:

Test statistic 0.000

Degrees of freedom 0

Model Test Baseline Model:

| Test statistic | 485.154 |
| --- | --- |
| Degrees of freedom | 5 |
| P-value | 0.000 |
| User Model versus Baseline Model: |  |
| Comparative Fit Index (CFI) | 1.000 |
| Tucker-Lewis Index (TLI) | 1.000 |
| Loglikelihood and Information Criteria: |  |
| Loglikelihood user model (H0) | -420.400 |
| Loglikelihood unrestricted model (H1) | -420.400 |
| Akaike (AIC) | 852.800 |

Bayesian (BIC) 876.150

Sample-size adjusted Bayesian (BIC) 857.115

Root Mean Square Error of Approximation:

RMSEA 0.000

90 Percent confidence interval - lower 0.000

90 Percent confidence interval - upper 0.000

P-value RMSEA <= 0.05 NA

Standardized Root Mean Square Residual:

SRMR 0.000

Parameter Estimates:

Standard errors Standard

Information Expected

Information saturated (h1) model Structured

| Regressions:  floss ~ | Estimate | Std.Err | z-value | P(>\|z\|) | Std.lv | Std.all |
| --- | --- | --- | --- | --- | --- | --- |
| intention | 0.082 | 0.016 | 5.048 | 0.000 | 0.082 | 0.253 |
| bp | 1.044 | 0.083 | 12.637 | 0.000 | 1.044 | 0.683 |
| mi | 0.001 | 0.003 | 0.465 | 0.642 | 0.001 | 0.013 |
| int_bp | -0.061 | 0.017 | -3.587 | 0.000 | -0.061 | -0.138 |
| int_mi | -0.001 | 0.001 | -1.592 | 0.111 | -0.001 | -0.044 |
| Variances: |  |  |  |  |  |  |
| Estimate | | Std.Err | z-value | P(>\|z\|) | Std.lv | Std.all |
| .floss 0.597 | | 0.044 | 13.454 | 0.000 | 0.597 | 0.262 |

Appendix C - 4: Model using Inhibit

- process (data, y = "floss", x = "intention", w ="bp", z = "inh", model = 2, jn = 1)

********************* PROCESS for R Version 4.0.1 ********************* Written by Andrew F. Hayes, Ph.D. [www.afhayes.com](http://www.afhayes.com/)

Documentation available in Hayes (2022). [www.guilford.com/p/hayes3](http://www.guilford.com/p/hayes3)

*********************************************************************** Model : 2

Y : floss

X : intention W : bp

Z : inh Sample size: 362

*********************************************************************** Outcome Variable: floss

Model Summary:

| R  0.8588 | R-sq  0.7375 | MSE  0.6091 | F  200.0412 | df1  5.0000 | df2  356.0000 | p  0.0000 |
| --- | --- | --- | --- | --- | --- | --- |
| Model: | coeff | se | t | p | LLCI | ULCI |
| constant | 3.3551 | 0.0693 | 48.4404 | 0.0000 | 3.2189 | 3.4913 |
| intention | 0.0810 | 0.0165 | 4.9120 | 0.0000 | 0.0486 | 0.1134 |
| bp | 1.0489 | 0.0833 | 12.5956 | 0.0000 | 0.8851 | 1.2127 |
| Int_1 | -0.0617 | 0.0171 | -3.6132 | 0.0003 | -0.0953 | -0.0281 |
| inh | -0.0037 | 0.0145 | -0.2577 | 0.7968 | -0.0321 | 0.0247 |
| Int_2 | -0.0042 | 0.0031 | -1.3571 | 0.1756 | -0.0103 | 0.0019 |

Product terms key:

Int_1 : intention x bp Int_2 : intention x inh

Test(s) of highest order unconditional interaction(s):

|  | R2-chng | F | df1 | df2 | p |
| --- | --- | --- | --- | --- | --- |
| X*W | 0.0096 | 13.0551 | 1.0000 | 356.0000 | 0.0003 |
| X*Z | 0.0014 | 1.8418 | 1.0000 | 356.0000 | 0.1756 |
| BOTH | 0.0104 | 7.0762 | 2.0000 | 356.0000 | 0.0010 |

Focal predictor: intention (X) Moderator: bp (W) Moderator: inh (Z)

Conditional effects of the focal predictor at values of the moderator(s):

| bp | inh | effect | se | t | p | LLCI | ULCI |
| --- | --- | --- | --- | --- | --- | --- | --- |
| -0.8953 | -2.8011 | 0.1480 | 0.0161 | 9.1986 | 0.0000 | 0.1163 | 0.1796 |

| -0.8953 | -0.8011 | 0.1396 | 0.0137 | 10.2032 | 0.0000 | 0.1127 | 0.1665 |
| --- | --- | --- | --- | --- | --- | --- | --- |
| -0.8953 | 3.1989 | 0.1228 | 0.0165 | 7.4378 | 0.0000 | 0.0904 | 0.1553 |
| -0.4032 | -2.8011 | 0.1176 | 0.0155 | 7.6110 | 0.0000 | 0.0872 | 0.1480 |
| -0.4032 | -0.8011 | 0.1092 | 0.0133 | 8.2290 | 0.0000 | 0.0831 | 0.1353 |
| -0.4032 | 3.1989 | 0.0925 | 0.0167 | 5.5283 | 0.0000 | 0.0596 | 0.1254 |
| 1.5469 | -2.8011 | -0.0028 | 0.0394 | -0.0702 | 0.9441 | -0.0802 | 0.0746 |
| 1.5469 | -0.8011 | -0.0111 | 0.0390 | -0.2856 | 0.7754 | -0.0879 | 0.0656 |
| 1.5469 | 3.1989 | -0.0279 | 0.0412 | -0.6772 | 0.4987 | -0.1089 | 0.0531 |

******************** ANALYSIS NOTES AND ERRORS ************************ Level of confidence for all confidence intervals in output: 95

W values in conditional tables are the 16th, 50th, and 84th percentiles. Z values in conditional tables are the 16th, 50th, and 84th percentiles.

- data$int_bp <- data$intention * data$bp
- data$int_inh <- data$intention * data$inh
- tst_model <- 'floss ~ intention+bp+inh+int_bp+int_inh'
- tst_fit <- sem(tst_model, data = data)
- summary(tst_fit, fit.measures=TRUE, standardized=TRUE) lavaan 0.6-12 ended normally after 1 iterations

Estimator ML

Optimization method NLMINB

Number of model parameters 6

Number of observations 362

Model Test User Model:

Test statistic 0.000

Degrees of freedom 0

Model Test Baseline Model:

| Test statistic | 484.181 |
| --- | --- |
| Degrees of freedom | 5 |
| P-value | 0.000 |
| User Model versus Baseline Model: |  |
| Comparative Fit Index (CFI) | 1.000 |
| Tucker-Lewis Index (TLI) | 1.000 |
| Loglikelihood and Information Criteria: |  |
| Loglikelihood user model (H0) | -420.887 |
| Loglikelihood unrestricted model (H1) | -420.887 |
| Akaike (AIC) | 853.774 |

Bayesian (BIC) 877.124

Sample-size adjusted Bayesian (BIC) 858.089

Root Mean Square Error of Approximation:

RMSEA 0.000

90 Percent confidence interval - lower 0.000

90 Percent confidence interval - upper 0.000

P-value RMSEA <= 0.05 NA

Standardized Root Mean Square Residual:

SRMR 0.000

Parameter Estimates:

Standard errors Standard

Information Expected

Information saturated (h1) model Structured

| Regressions:  floss ~ | Estimate | Std.Err | z-value | P(>\|z\|) | Std.lv | Std.all |
| --- | --- | --- | --- | --- | --- | --- |
| intention | 0.081 | 0.016 | 4.953 | 0.000 | 0.081 | 0.249 |
| bp | 1.049 | 0.083 | 12.701 | 0.000 | 1.049 | 0.686 |
| inh | -0.004 | 0.014 | -0.260 | 0.795 | -0.004 | -0.007 |
| int_bp | -0.062 | 0.017 | -3.644 | 0.000 | -0.062 | -0.140 |
| int_inh | -0.004 | 0.003 | -1.369 | 0.171 | -0.004 | -0.037 |
| Variances: |  |  |  |  |  |  |
| Estimate | | Std.Err | z-value | P(>\|z\|) | Std.lv | Std.all |
| .floss 0.599 | | 0.045 | 13.454 | 0.000 | 0.599 | 0.262 |

Appendix C - 5: Model using Shift

- process (data, y = "floss", x = "intention", w ="bp", z = "shi", model = 2, jn = 1)

********************* PROCESS for R Version 4.0.1 ********************* Written by Andrew F. Hayes, Ph.D. [www.afhayes.com](http://www.afhayes.com/)

Documentation available in Hayes (2022). [www.guilford.com/p/hayes3](http://www.guilford.com/p/hayes3)

*********************************************************************** Model : 2

Y : floss

X : intention W : bp

Z : shi Sample size: 362

*********************************************************************** Outcome Variable: floss

Model Summary:

| R  0.8606 | R-sq  0.7406 | MSE  0.6018 | F  203.3129 | df1  5.0000 | df2  356.0000 | p  0.0000 |
| --- | --- | --- | --- | --- | --- | --- |
| Model: | coeff | se | t | p | LLCI | ULCI |
| constant | 3.3526 | 0.0689 | 48.6653 | 0.0000 | 3.2172 | 3.4881 |
| intention | 0.0809 | 0.0164 | 4.9501 | 0.0000 | 0.0488 | 0.1131 |
| bp | 1.0446 | 0.0827 | 12.6239 | 0.0000 | 0.8819 | 1.2073 |
| Int_1 | -0.0601 | 0.0169 | -3.5466 | 0.0004 | -0.0934 | -0.0268 |
| shi | 0.0075 | 0.0167 | 0.4504 | 0.6527 | -0.0253 | 0.0404 |
| Int_2 | -0.0085 | 0.0035 | -2.4265 | 0.0157 | -0.0154 | -0.0016 |

Product terms key:

Int_1 : intention x bp Int_2 : intention x shi

Test(s) of highest order unconditional interaction(s):

|  | R2-chng | F | df1 | df2 | p |
| --- | --- | --- | --- | --- | --- |
| X*W | 0.0092 | 12.5781 | 1.0000 | 356.0000 | 0.0004 |
| X*Z | 0.0043 | 5.8878 | 1.0000 | 356.0000 | 0.0157 |
| BOTH | 0.0131 | 8.9847 | 2.0000 | 356.0000 | 0.0002 |

Focal predictor: intention (X) Moderator: bp (W) Moderator: shi (Z)

Conditional effects of the focal predictor at values of the moderator(s):

| bp | shi | effect | se | t | p | LLCI | ULCI |
| --- | --- | --- | --- | --- | --- | --- | --- |
| -0.8953 | -2.3840 | 0.1550 | 0.0156 | 9.9169 | 0.0000 | 0.1243 | 0.1857 |

| -0.8953 | -0.3840 | 0.1380 | 0.0134 | 10.2888 | 0.0000 | 0.1116 | 0.1644 |
| --- | --- | --- | --- | --- | --- | --- | --- |
| -0.8953 | 2.6160 | 0.1125 | 0.0163 | 6.8822 | 0.0000 | 0.0804 | 0.1447 |
| -0.4032 | -2.3840 | 0.1254 | 0.0152 | 8.2372 | 0.0000 | 0.0955 | 0.1554 |
| -0.4032 | -0.3840 | 0.1084 | 0.0131 | 8.2931 | 0.0000 | 0.0827 | 0.1341 |
| -0.4032 | 2.6160 | 0.0829 | 0.0162 | 5.1094 | 0.0000 | 0.0510 | 0.1149 |
| 1.5469 | -2.3840 | 0.0082 | 0.0394 | 0.2093 | 0.8343 | -0.0692 | 0.0857 |
| 1.5469 | -0.3840 | -0.0088 | 0.0388 | -0.2258 | 0.8215 | -0.0850 | 0.0675 |
| 1.5469 | 2.6160 | -0.0342 | 0.0402 | -0.8518 | 0.3949 | -0.1133 | 0.0448 |

******************** ANALYSIS NOTES AND ERRORS ************************ Level of confidence for all confidence intervals in output: 95

W values in conditional tables are the 16th, 50th, and 84th percentiles. Z values in conditional tables are the 16th, 50th, and 84th percentiles.

- data$int_bp <- data$intention * data$bp
- data$int_shi <- data$intention * data$shi
- tst_model <- 'floss ~ intention+bp+shi+int_bp+int_shi'
- tst_fit <- sem(tst_model, data = data)
- summary(tst_fit, fit.measures=TRUE, standardized=TRUE) lavaan 0.6-12 ended normally after 1 iterations

Estimator ML

Optimization method NLMINB

Number of model parameters 6

Number of observations 362

Model Test User Model:

Test statistic 0.000

Degrees of freedom 0

Model Test Baseline Model:

| Test statistic | 488.521 |
| --- | --- |
| Degrees of freedom | 5 |
| P-value | 0.000 |
| User Model versus Baseline Model: |  |
| Comparative Fit Index (CFI) | 1.000 |
| Tucker-Lewis Index (TLI) | 1.000 |
| Loglikelihood and Information Criteria: |  |
| Loglikelihood user model (H0) | -418.717 |
| Loglikelihood unrestricted model (H1) | -418.717 |
| Akaike (AIC) | 849.433 |

Bayesian (BIC) 872.783

Sample-size adjusted Bayesian (BIC) 853.748

Root Mean Square Error of Approximation:

RMSEA 0.000

90 Percent confidence interval - lower 0.000

90 Percent confidence interval - upper 0.000

P-value RMSEA <= 0.05 NA

Standardized Root Mean Square Residual:

SRMR 0.000

Parameter Estimates:

Standard errors Standard

Information Expected

Information saturated (h1) model Structured

| Regressions:  floss ~ | Estimate | Std.Err | z-value | P(>\|z\|) | Std.lv | Std.all |
| --- | --- | --- | --- | --- | --- | --- |
| intention | 0.081 | 0.016 | 4.992 | 0.000 | 0.081 | 0.249 |
| bp | 1.045 | 0.082 | 12.730 | 0.000 | 1.045 | 0.683 |
| shi | 0.008 | 0.017 | 0.454 | 0.650 | 0.008 | 0.012 |
| int_bp | -0.060 | 0.017 | -3.576 | 0.000 | -0.060 | -0.136 |
| int_shi | -0.008 | 0.003 | -2.447 | 0.014 | -0.008 | -0.066 |
| Variances: |  |  |  |  |  |  |
| Estimate | | Std.Err | z-value | P(>\|z\|) | Std.lv | Std.all |
| .floss 0.592 | | 0.044 | 13.454 | 0.000 | 0.592 | 0.259 |

Appendix C - 6: Model using Emotional Control

- process (data, y = "floss", x = "intention", w ="bp", z = "emo", model = 2, jn = 1)

********************* PROCESS for R Version 4.0.1 ********************* Written by Andrew F. Hayes, Ph.D. [www.afhayes.com](http://www.afhayes.com/)

Documentation available in Hayes (2022). [www.guilford.com/p/hayes3](http://www.guilford.com/p/hayes3)

*********************************************************************** Model : 2

Y : floss

X : intention W : bp

Z : emo Sample size: 362

*********************************************************************** Outcome Variable: floss

Model Summary:

| R  0.8610 | R-sq  0.7412 | MSE  0.6004 | F  203.9655 | df1  5.0000 | df2  356.0000 | p  0.0000 |
| --- | --- | --- | --- | --- | --- | --- |
| Model: | coeff | se | t | p | LLCI | ULCI |
| constant | 3.3564 | 0.0687 | 48.8360 | 0.0000 | 3.2212 | 3.4915 |
| intention | 0.0811 | 0.0163 | 4.9693 | 0.0000 | 0.0490 | 0.1132 |
| bp | 1.0453 | 0.0829 | 12.6162 | 0.0000 | 0.8823 | 1.2082 |
| Int_1 | -0.0623 | 0.0169 | -3.6812 | 0.0003 | -0.0955 | -0.0290 |
| emo | 0.0021 | 0.0097 | 0.2222 | 0.8243 | -0.0169 | 0.0212 |
| Int_2 | -0.0052 | 0.0020 | -2.6327 | 0.0088 | -0.0091 | -0.0013 |

Product terms key:

Int_1 : intention x bp Int_2 : intention x emo

Test(s) of highest order unconditional interaction(s):

|  | R2-chng | F | df1 | df2 | p |
| --- | --- | --- | --- | --- | --- |
| X*W | 0.0098 | 13.5513 | 1.0000 | 356.0000 | 0.0003 |
| X*Z | 0.0050 | 6.9313 | 1.0000 | 356.0000 | 0.0088 |
| BOTH | 0.0140 | 9.6460 | 2.0000 | 356.0000 | 0.0001 |

Focal predictor: intention (X) Moderator: bp (W) Moderator: emo (Z)

Conditional effects of the focal predictor at values of the moderator(s):

| bp | | emo | | effect | | se | | t | | p | | LLCI | | ULCI | |
| --- | --- | --- | --- | --- | --- | --- | --- | --- | --- | --- | --- | --- | --- | --- | --- |
| -0.8953 | | -4.2735 | | 0.1592 | | 0.0161 | | 9.8898 | | 0.0000 | | 0.1275 | | 0.1908 | |
| -0.8953 | | -1.2735 | | 0.1435 | | 0.0137 | | 10.4783 | | 0.0000 | | 0.1166 | | 0.1705 | |
| -0.8953 | | 4.7265 | | 0.1122 | | 0.0160 | | 6.9968 | | 0.0000 | | 0.0807 | | 0.1437 | |
| -0.4032 | | -4.2735 | | 0.1286 | | 0.0155 | | 8.2752 | | 0.0000 | | 0.0980 | | 0.1591 | |
| -0.4032 | | -1.2735 | | 0.1129 | | 0.0133 | | 8.5006 | | 0.0000 | | 0.0868 | | 0.1390 | |
| -0.4032 | | 4.7265 | | 0.0816 | | 0.0161 | | 5.0687 | | 0.0000 | | 0.0499 | | 0.1132 | |
| 1.5469 | | -4.2735 | | 0.0071 | | 0.0392 | | 0.1818 | | 0.8558 | | -0.0699 | | 0.0841 | |
| 1.5469 | | -1.2735 | | -0.0085 | | 0.0387 | | -0.2211 | | 0.8252 | | -0.0846 | | 0.0675 | |
| 1.5469 | | 4.7265 | | -0.0399 | | 0.0404 | | -0.9882 | | 0.3237 | | -0.1192 | | 0.0395 | |

******************** ANALYSIS NOTES AND ERRORS ************************ Level of confidence for all confidence intervals in output: 95

W values in conditional tables are the 16th, 50th, and 84th percentiles. Z values in conditional tables are the 16th, 50th, and 84th percentiles.

- data$int_bp <- data$intention * data$bp
- data$int_emo <- data$intention * data$emo
- tst_model <- 'floss ~ intention+bp+emo+int_bp+int_emo'
- tst_fit <- sem(tst_model, data = data)
- summary(tst_fit, fit.measures=TRUE, standardized=TRUE) lavaan 0.6-12 ended normally after 1 iterations

Estimator ML

Optimization method NLMINB

Number of model parameters 6

Number of observations 362

Model Test User Model:

Test statistic 0.000

Degrees of freedom 0

Model Test Baseline Model:

| Test statistic | 489.381 |
| --- | --- |
| Degrees of freedom | 5 |
| P-value | 0.000 |
| User Model versus Baseline Model: |  |
| Comparative Fit Index (CFI) | 1.000 |
| Tucker-Lewis Index (TLI) | 1.000 |
| Loglikelihood and Information Criteria: |  |
| Loglikelihood user model (H0) | -418.287 |
| Loglikelihood unrestricted model (H1) | -418.287 |
| Akaike (AIC) | 848.574 |

Bayesian (BIC) 871.924

Sample-size adjusted Bayesian (BIC) 852.889

Root Mean Square Error of Approximation:

RMSEA 0.000

90 Percent confidence interval - lower 0.000

90 Percent confidence interval - upper 0.000

P-value RMSEA <= 0.05 NA

Standardized Root Mean Square Residual:

SRMR 0.000

Parameter Estimates:

Standard errors Standard

Information Expected

Information saturated (h1) model Structured

| Regressions:  floss ~ | Estimate | Std.Err | z-value | P(>\|z\|) | Std.lv | Std.all |
| --- | --- | --- | --- | --- | --- | --- |
| intention | 0.081 | 0.016 | 5.011 | 0.000 | 0.081 | 0.250 |
| bp | 1.045 | 0.082 | 12.722 | 0.000 | 1.045 | 0.684 |
| emo | 0.002 | 0.010 | 0.224 | 0.823 | 0.002 | 0.006 |
| int_bp | -0.062 | 0.017 | -3.712 | 0.000 | -0.062 | -0.141 |
| int_emo | -0.005 | 0.002 | -2.655 | 0.008 | -0.005 | -0.071 |
| Variances: |  |  |  |  |  |  |
| Estimate | | Std.Err | z-value | P(>\|z\|) | Std.lv | Std.all |
| .floss 0.590 | | 0.044 | 13.454 | 0.000 | 0.590 | 0.259 |

Appendix C - 7: Model using Self-Monitor

- process (data, y = "floss", x = "intention", w ="bp", z = "sel", model = 2, jn = 1)

********************* PROCESS for R Version 4.0.1 ********************* Written by Andrew F. Hayes, Ph.D. [www.afhayes.com](http://www.afhayes.com/)

Documentation available in Hayes (2022). [www.guilford.com/p/hayes3](http://www.guilford.com/p/hayes3)

*********************************************************************** Model : 2

Y : floss

X : intention W : bp

Z : sel Sample size: 362

*********************************************************************** Outcome Variable: floss

Model Summary:

| R  0.8588 | R-sq  0.7375 | MSE  0.6090 | F  200.0680 | df1  5.0000 | df2  356.0000 | p  0.0000 |
| --- | --- | --- | --- | --- | --- | --- |
| Model: | coeff | se | t | p | LLCI | ULCI |
| constant | 3.3532 | 0.0692 | 48.4598 | 0.0000 | 3.2171 | 3.4892 |
| intention | 0.0817 | 0.0164 | 4.9666 | 0.0000 | 0.0493 | 0.1140 |
| bp | 1.0466 | 0.0833 | 12.5690 | 0.0000 | 0.8828 | 1.2104 |
| Int_1 | -0.0610 | 0.0170 | -3.5805 | 0.0004 | -0.0944 | -0.0275 |
| sel | -0.0022 | 0.0197 | -0.1096 | 0.9128 | -0.0409 | 0.0366 |
| Int_2 | -0.0056 | 0.0041 | -1.3725 | 0.1708 | -0.0137 | 0.0024 |

Product terms key:

Int_1 : intention x bp Int_2 : intention x sel

Test(s) of highest order unconditional interaction(s):

|  | R2-chng | F | df1 | df2 | p |
| --- | --- | --- | --- | --- | --- |
| X*W | 0.0095 | 12.8200 | 1.0000 | 356.0000 | 0.0004 |
| X*Z | 0.0014 | 1.8837 | 1.0000 | 356.0000 | 0.1708 |
| BOTH | 0.0105 | 7.0965 | 2.0000 | 356.0000 | 0.0010 |

Focal predictor: intention (X) Moderator: bp (W) Moderator: sel (Z)

Conditional effects of the focal predictor at values of the moderator(s):

| bp | sel | effect | se | t | p | LLCI | ULCI |
| --- | --- | --- | --- | --- | --- | --- | --- |
| -0.8953 | -2.4696 | 0.1501 | 0.0169 | 8.8576 | 0.0000 | 0.1168 | 0.1835 |

| -0.8953 | -0.4696 | 0.1389 | 0.0136 | 10.2227 | 0.0000 | 0.1122 | 0.1656 |
| --- | --- | --- | --- | --- | --- | --- | --- |
| -0.8953 | 2.5304 | 0.1221 | 0.0168 | 7.2747 | 0.0000 | 0.0891 | 0.1551 |
| -0.4032 | -2.4696 | 0.1201 | 0.0164 | 7.3114 | 0.0000 | 0.0878 | 0.1524 |
| -0.4032 | -0.4696 | 0.1089 | 0.0132 | 8.2306 | 0.0000 | 0.0829 | 0.1349 |
| -0.4032 | 2.5304 | 0.0921 | 0.0168 | 5.4668 | 0.0000 | 0.0589 | 0.1252 |
| 1.5469 | -2.4696 | 0.0013 | 0.0398 | 0.0315 | 0.9749 | -0.0770 | 0.0795 |
| 1.5469 | -0.4696 | -0.0100 | 0.0390 | -0.2560 | 0.7981 | -0.0866 | 0.0666 |
| 1.5469 | 2.5304 | -0.0268 | 0.0409 | -0.6556 | 0.5125 | -0.1072 | 0.0536 |

******************** ANALYSIS NOTES AND ERRORS ************************ Level of confidence for all confidence intervals in output: 95

W values in conditional tables are the 16th, 50th, and 84th percentiles. Z values in conditional tables are the 16th, 50th, and 84th percentiles.

- data$int_bp <- data$intention * data$bp
- data$int_sel <- data$intention * data$sel
- tst_model <- 'floss ~ intention+bp+sel+int_bp+int_sel'
- tst_fit <- sem(tst_model, data = data)
- summary(tst_fit, fit.measures=TRUE, standardized=TRUE) lavaan 0.6-12 ended normally after 1 iterations

Estimator ML

Optimization method NLMINB

Number of model parameters 6

Number of observations 362

Model Test User Model:

Test statistic 0.000

Degrees of freedom 0

Model Test Baseline Model:

| Test statistic | 484.216 |
| --- | --- |
| Degrees of freedom | 5 |
| P-value | 0.000 |
| User Model versus Baseline Model: |  |
| Comparative Fit Index (CFI) | 1.000 |
| Tucker-Lewis Index (TLI) | 1.000 |
| Loglikelihood and Information Criteria: |  |
| Loglikelihood user model (H0) | -420.869 |
| Loglikelihood unrestricted model (H1) | -420.869 |
| Akaike (AIC) | 853.738 |

Bayesian (BIC) 877.088

Sample-size adjusted Bayesian (BIC) 858.053

Root Mean Square Error of Approximation:

RMSEA 0.000

90 Percent confidence interval - lower 0.000

90 Percent confidence interval - upper 0.000

P-value RMSEA <= 0.05 NA

Standardized Root Mean Square Residual:

SRMR 0.000

Parameter Estimates:

Standard errors Standard

Information Expected

Information saturated (h1) model Structured

| Regressions:  floss ~ | Estimate | Std.Err | z-value | P(>\|z\|) | Std.lv | Std.all |
| --- | --- | --- | --- | --- | --- | --- |
| intention | 0.082 | 0.016 | 5.008 | 0.000 | 0.082 | 0.252 |
| bp | 1.047 | 0.083 | 12.674 | 0.000 | 1.047 | 0.685 |
| sel | -0.002 | 0.020 | -0.110 | 0.912 | -0.002 | -0.003 |
| int_bp | -0.061 | 0.017 | -3.611 | 0.000 | -0.061 | -0.138 |
| int_sel | -0.006 | 0.004 | -1.384 | 0.166 | -0.006 | -0.037 |
| Variances: |  |  |  |  |  |  |
| Estimate | | Std.Err | z-value | P(>\|z\|) | Std.lv | Std.all |
| .floss 0.599 | | 0.045 | 13.454 | 0.000 | 0.599 | 0.262 |

Appendix D - Syntax Used to Generate Simple Slopes

# Splitting of the data

data$meanSplitBP <- ifelse((data$bp > 0), 1, 0) # Create splitting variable splitBpAnalysis <- split(data, data$meanSplitBP) # Split based on this variable highBP <- splitBpAnalysis$`1` # Create high and low groups

lowBP <- splitBpAnalysis$`0`

# Get the first and third quartiles for the moderators shi_summary <- summary(data$shi) # Get quartile summaries emo_summary <- summary(data$emo)

firstQ_shi <- shi_summary[[2]] # Create variable for first quartile value thirdQ_shi <- shi_summary[[5]] # Create variable for third quartile value firstQ_emo <- emo_summary[[2]] # Repeat for emotional control

thirdQ_emo <- emo_summary[[5]]

# Get the regression equation summaries for each BP group (high/low, shifting/emotional control)

reg_shi_lowBP <- summary(lm(floss ~ intention + shi + int_shi, data = lowBP)) reg_shi_highBP <- summary(lm(floss ~ intention + shi + int_shi, data = highBP)) reg_emo_lowBP <- summary(lm(floss ~ intention + emo + int_emo, data = lowBP)) reg_emo_highBP <- summary(lm(floss ~ intention + emo + int_emo, data = highBP))

# Calculate the simple slopes using the given data and linear algebra

# Each graph will have 4 lines, meaning 8 lines to be calculated in total

# The following will return the intercept and slope of these lines, allowing them to be plotted

## First, substitute the correct variables into the following lines - *IMPORTANT* ss_model <- reg_shi_lowBP # Dictate which EF to graph, and which level of BP (4 options)

ss_efLevel <- firstQ_shi # For each group, there are 2 lines based on EF. Pick which line to model

b_0 <- ss_model$coefficients[1] # Create b-weights from the model coefficients b_1 <- ss_model$coefficients[2]

b_2 <- ss_model$coefficients[3] b_3 <- ss_model$coefficients[4]

ss_intercept <- b_0 + (ss_efLevel*b_2) # Calculate the intercept ss_slope <- b_1 + (ss_efLevel*b_3) # Calculate the slope

# Generate simple slope code for plotting print(paste0("abline(", ss_intercept, ",", ss_slope, ")"))

## END OF SYNTAX

For the above example, the simple slope of lower shifting scores among those with low BP, the following code is returned:

abline(2.75878204161995,0.208284496412526)

Note that lower shifting scores indicate fewer problems with shifting, so "low shifting" is referred to as "high shifting capacity" in the article. The following is the output from all four regression models and the intercept and slope parameters generated by substituting first and third quartile values of the moderator into each model as per the above syntax.

####### MODEL 1 #######

summary(lm(floss ~ intention + shi + int_shi, data = lowBP)) Call:

lm(formula = floss ~ intention + shi + int_shi, data = lowBP)

Residuals:

Min 1Q Median 3Q Max

-1.9494 -0.6531 -0.1595 0.5255 3.4456

Coefficients:

|  | Estimate | Std. Error | t value | Pr(>\|t\|) |  |
| --- | --- | --- | --- | --- | --- |
| (Intercept) | 2.689061 | 0.075813 | 35.470 | < 2e-16 | *** |
| intention | 0.165941 | 0.015719 | 10.557 | < 2e-16 | *** |
| shi | -0.029246 | 0.033998 | -0.860 | 0.39062 |  |
| int_shi | -0.017762 | 0.006817 | -2.606 | 0.00981 | ** |
| --- |  |  |  |  |  |

Signif. codes: 0 ‘***’ 0.001 ‘**’ 0.01 ‘*’ 0.05 ‘.’ 0.1 ‘ ’ 1

Residual standard error: 0.9538 on 216 degrees of freedom Multiple R-squared: 0.3561, Adjusted R-squared: 0.3472 F-statistic: 39.82 on 3 and 216 DF, p-value: < 2.2e-16

Low Shifting: abline(2.75878204161995,0.208284496412526) High Shifting: abline(2.6417998439425,0.137237361688767)

####### MODEL 2 #######

summary(lm(floss ~ intention + shi + int_shi, data = highBP)) Call:

lm(formula = floss ~ intention + shi + int_shi, data = highBP)

Residuals:

Min 1Q Median 3Q Max

-2.6707 -0.5031 0.3234 0.3278 1.7223

Coefficients:

|  | Estimate | Std. Error | t value | Pr(>\|t\|) |  |
| --- | --- | --- | --- | --- | --- |
| (Intercept) | 4.056686 | 0.107884 | 37.602 | < 2e-16 | *** |
| intention | 0.129078 | 0.024383 | 5.294 | 4.61e-07 | *** |
| shi | -0.059539 | 0.033683 | -1.768 | 0.0793 | . |
| int_shi | 0.012721 | 0.007504 | 1.695 | 0.0923 | . |
| --- |  |  |  |  |  |

Signif. codes: 0 ‘***’ 0.001 ‘**’ 0.01 ‘*’ 0.05 ‘.’ 0.1 ‘ ’ 1

Residual standard error: 0.6689 on 138 degrees of freedom Multiple R-squared: 0.2015, Adjusted R-squared: 0.1841

F-statistic: 11.61 on 3 and 138 DF, p-value: 7.881e-07

Low Shifting: abline(4.19862442898883,0.0987517811079517) High Shifting: abline(3.96047002149675,0.149635856328451)

####### MODEL 3 #######

summary(lm(floss ~ intention + emo + int_emo, data = lowBP)) Call:

lm(formula = floss ~ intention + emo + int_emo, data = lowBP)

Residuals:

Min 1Q Median 3Q Max

-1.9637 -0.6354 -0.1737 0.5456 3.3069

Coefficients:

|  | Estimate | Std. Error | t value | Pr(>\|t\|) |  |
| --- | --- | --- | --- | --- | --- |
| (Intercept) | 2.710153 | 0.074266 | 36.493 | < 2e-16 | *** |
| intention | 0.171020 | 0.015366 | 11.130 | < 2e-16 | *** |
| emo | -0.047470 | 0.017995 | -2.638 | 0.00895 | ** |
| int_emo | -0.014600 | 0.003517 | -4.151 | 4.77e-05 | *** |
| --- |  |  |  |  |  |

Signif. codes: 0 ‘***’ 0.001 ‘**’ 0.01 ‘*’ 0.05 ‘.’ 0.1 ‘ ’ 1

Residual standard error: 0.9334 on 216 degrees of freedom Multiple R-squared: 0.3834, Adjusted R-squared: 0.3748 F-statistic: 44.76 on 3 and 216 DF, p-value: < 2.2e-16

Low Emotional Control: abline(2.86554394436222,0.218812711786841) High Emotional Control: abline(2.58072609463706,0.131212942102365)

####### MODEL 4 #######

summary(lm(floss ~ intention + emo + int_emo, data = highBP)) Call:

lm(formula = floss ~ intention + emo + int_emo, data = highBP)

Residuals:

Min 1Q Median 3Q Max

| -2.6642 -0.5240 0.3170 | 0.3484 | 2.0108 |  |
| --- | --- | --- | --- |
| Coefficients: |  |  |  |
| Estimate | Std. Error | t value | Pr(>\|t\|) |
| (Intercept) 4.0339174 | 0.1103404 | 36.559 | < 2e-16 *** |
| intention 0.1343941 | 0.0250749 | 5.360 | 3.41e-07 *** |
| emo 0.0109135 | 0.0306869 | 0.356 | 0.723 |
| int_emo -0.0009684 | 0.0068838 | -0.141 | 0.888 |

---

Signif. codes: 0 ‘***’ 0.001 ‘**’ 0.01 ‘*’ 0.05 ‘.’ 0.1 ‘ ’ 1

Residual standard error: 0.6764 on 138 degrees of freedom Multiple R-squared: 0.1835, Adjusted R-squared: 0.1657

F-statistic: 10.34 on 3 and 138 DF, p-value: 3.514e-06

Low Emotional Control: abline(3.99819211111916,0.137564124362703) High Emotional Control: abline(4.063673341145,0.131753798957459)
